# *Leishmania donovani* Inhibitor of Serine Peptidases 2 Mediated Inhibition of Lectin Pathway and Upregulation of C5aR Signaling Promote Parasite Survival inside Host

**DOI:** 10.3389/fimmu.2018.00063

**Published:** 2018-01-29

**Authors:** Sudha Verma, Abhishek Mandal, Md. Yousuf Ansari, Ajay Kumar, Kumar Abhishek, Ayan Kumar Ghosh, Ashish Kumar, Vinod Kumar, Sushmita Das, Pradeep Das

**Affiliations:** ^1^Department of Molecular Biology, Rajendra Memorial Research Institute of Medical Sciences (ICMR), Patna, India; ^2^MM College of Pharmacy, Maharishi Markandeshwar University, Ambala, India; ^3^Department of Microbiology, All India Institute of Medical Sciences, Patna, India

**Keywords:** ISP2, MBL-associated serine proteases, membrane attacking complex, serine proteases, *Leishmania*, PI3K

## Abstract

*Leishmania donovani*, the causative agent of Indian visceral leishmaniasis has to face several barriers of the immune system inside the mammalian host for its survival. The complement system is one of the first barriers and consists of a well-balanced network of proteases including S1A family serine proteases (SPs). Inhibitor of serine peptidases (ISPs) is considered as inhibitor of S1A family serine peptidases and is reported to be present in trypanosomes, including *Leishmania*. In our previous study, we have deciphered the role of ISPs [LdISP1 and *L. donovani* inhibitor of serine peptidases 2 (LdISP2)] in the survival of *L. donovani* inside the sandfly midgut. However, the role of theses ISPs in the survival of *L. donovani* inside mammalian host still remains elusive. In the present study, we have deciphered the inhibitory effect of LdISPs on the host complement S1A serine peptidases, such as C1r/C1s and MASP1/MASP2. Our study suggested that although both rLdISP1 and rLdISP2 inferred strong interaction with C1complex and MBL-associated serine proteases (MASPs) but rLdISP2 showed the stronger inhibitory effect on MASP2 than rLdISP1. Moreover, we found that rLdISP2 significantly reduces the formation of C3, C5 convertase, and membrane attacking complex (MAC) by lectin pathway (LP) resulting in significant reduction in serum mediated lysis of the parasites. The role of LdISP2 on neutrophil elastase-mediated C5aR signaling was also evaluated. Notably, our results showed that infection of macrophages with ISP2-overexpressed *Leishmania* parasites significantly induces the expression of C5aR both at the transcript and translational level. Simultaneously, infection with ISP2KD parasites results in downregulation of host PI3K/AKT phosphorylation and increased in IL-12 production. Taken together, our findings clearly suggest that LdISP2 promotes parasite survival inside host by inhibiting MAC formation and complement-mediated lysis *via* LP and by upregulation of C5aR signaling.

## Introduction

Leishmaniasis is one of the most important infectious diseases caused by protozoan parasite *Leishmania* sp., and transmitted to the human or other mammalian hosts by sandfly vector, affecting ~0.7 million of people worldwide (1). When an infective sand fly bites uninfected (UI) individual *Leishmania* parasites are transmitted to the host where they have to evade the innate immune response of the host for its persistence and establishment of infection (2, 3).

The complement system is a self-propagating proteolytic cascade of proteins in form of innate immunity. It acts as one of the first barriers of the immune system for the parasite evasion inside the host, whereas evading the complement attacks is a key determinant for the parasite survival within their hosts (4). The three major pathways of complement system include classical pathway (CP), lectin pathway (LP), and alternative pathways (AP). It consists of a well-balanced network of circulating and cell-surface-bound proteins, which serve as substrates, enzymes, or modulators of a hierarchical series of extracellular proteolytic cascades (5). Serine proteases (SPs) are key components of the complement system present in the circulation as zymogens (6). C1r, C1s, and MBL-associated serine proteases (MASPs) have been reported as some of the SPs of classical and LP, respectively, that belongs to the S1A family (7). MASPs (MASP1 and MASP2) are homologs of C1r and C1s, with identical SP domain organization like chymotrypsin (8).

Classical pathway activated by the formation of soluble antigen–antibody complex that induces the binding of the antibody molecule to the C1 component. C1 component in the serum is a macromolecule consists of C1q and two molecules each of C1r and C1s, held together in a complex known as C1 complex (C1qr2s2) (9). However, LP activated by binding of mannose-binding lectin (MBL) to mannose present on the surface of parasite or microorganism (10). When MBL binds to the surface of the pathogen, it gets activated and binds with mannose-associated SPs, MASP1 and MASP2 to activate them (4). C1qr2s2 and MASP2 proteases are responsible for the conversion of the recognition signal into an enzymatic one by autoactivation and cleavage of C4 and C2 molecule (8). After induction with appropriate stimuli, this protease activates each other in a cascade-like manner resulting in C3 convertase (C3c) formation by classical/LP (C4b2a). AP are activated by the pathogen biomolecules, resulting in auto-hydrolysis of circulating C3 molecule that leads to the formation of C3c (C3bBb) (10). Re-assembled C3c then cleaves C3 molecules into C3a and C3b, in which C3b binds nearby surfaces providing platform for C5 convertase (C5c) formation *via* classical/LP (C4b2aC3b) and AP (C3bBb3b), (9). C5c cleaves C5 molecule into C5a and C5b, the later then binds to the pathogen surface to form an anchor, together with C6, C7, and C8 and form membrane attacking complex (MAC) with several C9 molecules resulting in parasite lysis (10).

Complement system function should be highly regulated because any disturbance in the delicate balance may results in increased susceptibility to infections. Deficiency of any key component of the pathway results in impaired complement activation and inefficient lysis of the parasites (11, 12). Trypanosomes or *Leishmania* uses different strategies/molecules to evade the host complement attack for its successful invasion of the host (3). It was reported that calreticulin (CRT) of *Trypanosoma cruzi* (*T. cruzi*) inhibits activation of classical and LP (13–15) of the host whereas gp58/68 protein of *T. cruzi* inhibits the formation of C3c by AP (16). Moreover, in *Leishmania major* it was reported that GP63, a metalloproteases inactivate C3b molecule to C3bi, prevent C3c formation and thereby complement-mediated lysis of the parasites (17). In another study, it has been reported that *L. major* metacyclic promastigotes prevent insertion of lytic C5b-9 MAC to their surface by a modified LPG, that is approximately twice as long as found on the surface of procyclic promastigotes and prevent complement-mediated lysis (18).

The presence of the inhibitor of serine peptidases (ISPs) has been reported to be present in *Leishmania donovani* (19) recently. ISPs are the known inhibitor of S1A family serine peptidases (20), the peptidases that are absent in the protozoan parasites. This parasite-derived ISP2 are known to protect the invading organism from degradation by the host-derived S1A peptidases, such as neutrophil elastase (NE), trypsin, and chymotrypsin (21). It has been reported earlier that *L. major* ISP2 promotes parasite survival by inhibition of NE (22). Van den Berg reported that NE cleaves C5aR receptor and thereby inactivates C5a induced signaling (23). However, the correlation between ISPs of *L. donovani* (LdISPs) and complement S1A peptidases has yet not been elucidated. The LdISPs might inhibit the SP of the complement channel and help the parasite to survive inside the host.

In the present study, we have observed the inhibitory effect of LdISPs on the host complement serine peptidases C1r, C1s, MASPs (MASP1/MASP2) and its affector cross road complement molecule, i.e., C3c and C5c formation. We have found that *L. donovani* inhibitor of serine peptidases 2 (LdISP2) inhibits MASP2 activity leading to the reduction in MAC formation and parasite lysis by LP. Further, we have assessed the role of LdISP2 on NE and C5aR-mediated PI3K/AKT signaling. Our study suggested that LdISP2 promotes parasite survival inside host by inhibiting MAC formation and complement-mediated lysis *via* LP and by upregulation of C5aR signaling.

## Materials and Methods

### Chemical and Reagents

THP1 cells, a human monocytic macrophage-like cell line was obtained from National Centre for Cell Science (Pune, Maharashtra, India). Mannan from *Saccharomyces cerevisiae*, penicillin G-sodium, streptomycin, human C4 molecule, anti-human C3b antibody, anti-human C4b antibody, NE from human leukocytes, substrate -*N*-α-Cbz-l-lysine thiobenzyl ester (Z-l-Lys-SBzl), VPR–7-amino-4 methyl coumarin, *N*-methoxy succinyl–Ala–Ala–Pro–Val-7-amino-4-methyl curamin, and DTNB [5,5-di thio-bis-(2 nitro-benzoic acid)] were purchased from Sigma-Aldrich (USA). Ficoll-Plaque Plus was purchased from GE Healthcare (Piscataway, NJ, USA). Human IgM and C1 complex were purchased from Merk-Millipore (US). M199 media and fetal bovine serum (FBS) were purchased from Invitrogen (USA) and Gibco (USA), respectively. C3c and C5c ELISA kits were purchased from Life Science, Inc. (USCN). Antibody against human C5aR, C5b-C9, PI3K, AKT, p-AKT, and p-PI3K were purchased from Santa Cruz Biotechnology (USA). Complement C1q antibody, anti-human MBL antibody, Factor B antibody against human were purchased from Abcam (Cambridge, UK). Secondary antibodies (HRP conjugated) were purchased from Jackson Laboratory (USA).

### Parasite Culture

*L. donovani* promastigotes (AG83:MHOM/IN/1983/AG83) were used in our experiments. ISP2KD and ISP2 overexpressed (ISP2OE) *Leishmania* parasites were prepared in our lab as described previously (24). All the cell lines promastigotes were maintained according to Mandal et al. (25) at 25°C in 25 cm^2^ flasks in fresh Medium-199 supplemented with 10% FBS. The ISP2KD and ISP2OE cells were maintained in M199 media supplemented with neomycin (G418) at 200 µg/ml concentration (24).

### Macrophage Cell Culture

THP1 cells, a human monocytic macrophage-like cell line was cultured in RPMI-1640 medium containing 10 mM HEPES, 1 mM sodium pyruvate supplemented with 4 mM NaHCO_3_, penicillin G-sodium (100 U/ml), streptomycin (100 mg/ml), and 10% (v/v) FBS. The cells were maintained in 5% CO_2_ incubator at 37°C in tissue culture flask (Nunc A/S Roskilde, Denmark). The cells were subcultured at every 3–4 days to maintain the confluency of cells. For experimental infection, THP1cells (~10^6^) were treated with 20 nM phorbol 12-myristate 13-acetate (PMA) (Sigma, St. Louis, MO, USA) and incubated for 24 h to become adherent and matured. Prior to infection, cells were washed with RPMI media without FBS to remove non-adherent cells.

Human mononuclear cells were isolated by the method as described by Singh et al. (26). Briefly, the peripheral blood of the healthy donor was collected in a heparinized tube. Isolated blood was processed immediately for mononuclear cell isolation by Ficoll-Paque density gradient method according to the manufacture instructions. The isolated monocytes were cultured (~1 × 10^6^ cells) in RPMI medium with 10% FBS in the presence of macrophage colony-stimulating factor (300 ng/ml) according to Mandal et al. (25). Washing was performed repeatedly to remove non- adherent cells and the cells were allowed to differentiate into human monocyte-derived macrophages (hMDM). Differentiated hMDM were harvested after 96 h for further infection study.

### *In Silico* Interaction of LdISP1 and LdISP2 with C1r, C1s, MASP1, and MASP2: Protein–Protein Interaction Studies

In our previous report, the robust homology models of LdISP1 and LdISP2 was used to monitor its interaction with trypsin and chymotrypsin (24). Here, we considered those putative models of ISP1 and ISP2 for further interaction study. First, the protein models of ISP1 and ISP2 were refined by Galaxy WEB server (27) and we have evaluated their reliability by SAVES server (ERRAT) (28). The refined models of ISP1 and ISP2 were further validated by Verify 3D. Finally ProSA-web server was used to evaluate the generated 3D structure of ISP1 and ISP2 for potential stability (29). The final refined protein model of LdISP1 and LdISP2 were used for the interaction study with the complement proteins C1r, C1s, MASP1, and MASP2. The PDB structure of the complement proteins of C1r (PDB: 1MD8_A), C1s (PDB: 1ELV_A), MASP1 (PDB: 3GOV_B), and MASP2 (PDB: 1Q3X_A) was taken from PDB database (3D structures) (30–33). The protein preparation of the selected protein structure was performed in Discovery Studio 2.5 (DSv2.5) with an application of a CHARMm force field (34). GRAMM (Global Range Molecular Matching)-X (Web Server v.1.2.0 program) was used for protein–protein docking (35). The generated complex model of proteins were further analyzed and visualized in the DSv2.5 for protein–protein interaction (24, 36).

### ELISA to Assess Interaction of rLdISP1 and rLdISP2 with C1 Complex and MASPs

ELISA was performed to study the interaction of rLdISP1 and rLdISP2 with C1 complex and MASPs according to Ferreira et al. (13) with minor modification. Briefly, ELISA plate was coated with either human IgM (10 µg/ml) or mannan from *S. cerevisiae* (5 µg/ml) in coating buffer (15 mM of NaCO_3_, 35 mM of NaHCO_3_, pH 9.6). Purified C1 complex (0–100 µg/ml) was diluted in PBS/1% w/v bovine serum albumin (BSA)/0.05% tween 20, whereas normal human serum (NHS) was diluted in veronal buffer saline (VBS) (145 mM/L NaCl, 1.8 m M/L sodium barbiturate, 2.8 mM barbiturate acid) in different dilution (1:100; 1:50; 1:10). Different concentration of C1 complex and serum was added to the antibody-coated plates or mannan-coated plates, respectively, incubate at 37°C for 2 h. Washing was performed throughout the assay with washing buffer, TBS (10 mM of NaCl)/0.05% Tween. Subsequently, after washing different concentration (0–50 µM) of rLdISP1 and rLdISP2 were added to the coated plates and incubated at 37°C for 2 h. Bound rLdISP1 and rLdISP2 was detected with the anti-ISP1 or anti-ISP2 antibody (1:500 dilutions) followed by addition of HRP conjugated anti-goat IgG-Ab and TMB substrate (3,3′,5,5′-tetramethylbenzidine).

### Enzymatic Assay

The enzymatic assay was performed to observe the inhibitory property of rLdISP1 and rLdISP2 on C1 complex, MASPs and NE activities. ELISA plate was coated either with human IgM or mannan. Purified C1 complex (100 µg/ml) was bound to the antibody-coated plate as discussed previously. Mannan-coated wells were bound with 100 µl of serum (1:10 v/v) diluted in serum dilution buffer (40 mM HEPES, 2 M NaCl, 10 mM CaCl_2_, pH 7.4) and incubated for 2 h at 37°C. C1 and serum bound wells were washed with washing buffer according to Presanis et al. (37). After washing, both C1 complex and MASPs-bound plate was incubated with or without rLdISP1/LdISP2 (0–50 µM) or C1-inhibitor (C1-inh) (10 nM) for 1 h. The C1 complex or mannan-coated plates without inhibitor were used as a control. Relative activity of C1 complex and MASP2 was tested by using the chromogenic substrate (100 µM) such as-N-α-Cbz-l-lysine thiobenzyl ester (Z-l-Lys-SBzl) and DTNB [5,5-di thio-bis-(2 nitro-benzoic acid)] according to Keizer et al. (38). The absorbance was recorded at 405 nm for every 10 min for 1 h. Simultaneously, MASP1 activity was analyzed by addition of MASP1 specific substrate VPR–7-amino-4 methyl to the MASPs-bound plate and incubated for 1 h at 37°C. Approximately, 100 µM of substrate diluted in 20 mM HEPES, 5 mM CaCl2, pH 8.5 (VB2 + B/T) buffer was used in the experiments. The fluorescent AMC group is released upon cleavage by active protease and the relative release of AMC was monitored over a time course using an excitation wavelength of 355 nm and an emission wavelength of 450 nm by spectroflurometer.

Additionally, the enzymatic assay was performed according to Morrisons et al. (39) to see the inhibitory effect of rLdISPs (rLdISP1 and rLdISP2) on NE. Briefly, NE (50 nM) was either left untreated or treated with inhibitor ecotin (1 µM) or rLdISP1/rLdISP2 (0.1–10 µM) in 100 mM Tris–HCl, pH 8.0, 2.5% (v/v) dimethyl sulfoxide and incubated in ice for 30 min. Subsequently, the appropriate chromogenic substrate, i.e., *N*-methoxy succinyl–Ala-Ala-Pro-Val-7-amino-4-methyl curamin (200 µM) was added to treated and untreated NE. Enzymatic hydrolysis of the substrate was monitored by spectrofluorometer by measuring the release of fluorescence (excitation 380 nm, emission at 460 nm). All the experiments were performed in triplicate, and the data expressed as means ± SD from three independent experiments.

### Measurement of C4b Formation by ELISA

To observe the inhibitory effect of rLdISPs on MASPs functional activity, C4 cleavage assay was performed in presence or absence of rLdISP1/rLdISP2 according to Ferreira et al. (13) with minor modification. Briefly, the microtitration plate was firstly coated with mannan as discussed previously. NHS was diluted (1:10 v/v) in BVB^+^ buffer (VBS containing 0.5 mM MgCl2·6H_2_O, 1.5 mM CaCl_2_, 0.05% tween-20, 1% BSA, pH 7.5). NHS pretreated with rLdISP1/rLdISP2 (0–50 µM) at 4°C for 1 h, or untreated NHS was added to the mannan-coated plates and incubated for 2 h at 37°C. The ELISA plate bound with serum-derived MASPs was used as a control. NHS pre-incubated with C1-inh (10 nM), the known inhibitor of classical and LP was used as the control inhibitor. C4 molecule (0.2 µg/ml) containing 20 mM HEPES, 140 mM NaCl, 5 mM CaCl_2_, pH 7.4, was added to the serum bound plate and incubated at 37°C for 2 h. Simultaneously, another plate was coated with C4b antibody (diluted in coating buffer) and incubated for 2 h. The cleaved C4 product was added to C4b antibody bound plate and further incubated for 2 h. The C4b antibody was added to the plate and incubated for another 2 h. The plates were washed in each step with TBS/0.05% Tween/5 mM of CaCl_2_, according to Presanis et al. (37). The bound C4b antibody was quantified by addition of HRP-conjugated secondary antibody followed by TMB substrate. Absorbance was recorded at 450 nm in an ELISA plate reader. Each experiment was done in triplicate and data are means ± SD from three independent experiments.

### ELISA for C3, C5c Assessment

Functional convertase assay was performed to monitor the C3c and C5c formation *via* all active complement pathway and LP individually. Serum was prepared according to the different experimental setup. Briefly, to study the involvement of all three complement pathways, NHS was firstly diluted (1:10 v/v) in modified veronal buffer (VB^2+^—4 mM of 5,5-diethylbarbituric acid sodium salt, 10 mM of NaCl, 2 mM of CaCl2, and 1 mM of MgCl2, pH 7.4). To determine the contribution of LP in C3c and C5c formation, lectin pathway-specific serum (LPSS) was prepared by incubating NHS (10% v/v) with “C1q antibody + Factor B antibody” (40). To block the formation of C3 and C5c formation *via* all pathways and individually by LP NHS or LPSS was incubated with “C1-inh + Factor B antibody” and “C1-inh” (10 nM), respectively, kept at 37°C for 2 h. The appropriate dilution of the antibody used in the experiment was standardized experimentally.

Further, to assess the inhibitory role of rLdISP1/rLdISP2 on convertase formation, the different group of serum was incubated with rLdISP1/rLdISP2 (0–50 µM) at 37°C for 2 h. Untreated NHS and LPSS were used as a control. *Leishmania* promastigotes (~2 × 10^6^) were processed according to Okroj et al. (41) for the convertase formation. Processed cells were added in the different group of untreated serum or serum pre-incubated with different inhibitors and kept in an incubator shaker at 30°C with shaking at 300 rpm for 5–10 min for C3 and C5c formation. Simultaneously, for the assessment of C3c, C5c formation C3 and C5c ELISA kits were used, respectively, to perform ELISA by using the processed serum sample. The experiments were performed in triplicate and data are means ± SD from three separate experiments.

### Complement-Mediated Functional Activity and Lysis Assay

The comparative study of the complement pathway activation by *Leishmania* parasites was detected by the deposition of C3b molecule on parasite surface and formation of MAC (Detail in SI) (11, 42). Additionally, LP activation assay was performed using rLdISP2 to analyze the relative MAC (C5b-9) formation and parasite lysis. For the LP activation, LPSS was used. Heat inactive LPSS, active LPSS, LPSS pretreated with C1-inh (10 nM), or LPSS pretreated with different concentration of rLdISP2 (5–50 µM) were used for this study. Briefly, for LP activation, *Leishmania* cells were first washed three times with ice-cold Gelatin HEPES buffer saline containing 2 mM CaCl_2_ and 0.5 mM MgCl_2_ (GHB^2+^). Two experimental set up was made, in which an equal number of *Leishmania* promastigotes (~1 × 10^7^) were taken. In one set, washed parasites were fixed in 4% paraformaldehyde and coated in ELISA plate for overnight at 4°C (12). The wells were washed with phosphate buffered saline (PBS-137 mM NaCl, 1.47 mM KH_2_PO_4_, 4.3 mM Na_2_HPO_4_, 2.7 mM KCl)/0.05% tween 20 (3 times) then blocked with 3% BSA in PBS for 2 h at room temperature. For LP activation, different group of LPSS were added into the well and incubate for 1 h. Active formation of MAC *via* each group of serum was detected by using anti-human C5b-9 complex antibody (1: 1,000 dilution), followed by incubation with HRP conjugated secondary antibody (1:5,000). In another set, parasites were either treated with heat inactive LPSS, active LPSS, LPSS pretreated with C1-inh (10 nM), LPSS pretreated with different concentration of rLdISP2 (5–50 µM). The treated parasites were used to analyze the complement-mediated lysis by counting the number of viable parasites in hemocytometer by trypan blue method. The parasites were stained with Giemsa for the microscopic examination of the different group of serum treated parasites.

### Infection of Macrophages with *L. donovani* Parasites

For infection experiment, equal number of THP1 and hMDM (~10^6^) were used for two experimental setups. In one set, the adhered THP1 cells and hMDM were infected with either wild-type (WT), ISP2KD, or ISP2OE *Leishmania* parasites at parasite/macrophage multiplicities of 10:1. Cultures were kept at 37°C in 5% CO_2_ for 12 h. The unbound parasites were removed by washing with RPMI without FBS. The cells were harvested (12 h after infection) from each group, washed with PBS and kept at −80°C for RNA and protein isolation. In other set, macrophages (THP1 and hMDM) were pretreated with wortmannin (200 nM) for 3 h and then infected with WT parasites. Untreated macrophages were infected with WT, ISP2KD, and ISP2OE parasites. Infected cells were kept in incubator and cells were harvested (after 12 h) for protein isolation and the supernatant was collected for ELISA and kept at −80°C. The parasite load was measured both in THP1 cells and hMDM at 24 h after infection by counting the number of intracellular amastigotes per 100 macrophages and the rate of infection was also analyzed in both cells (THP1 and hMDM) by counting the percent infected macrophages after Giemsa staining.

### Macrophage Treatment to NE

THP1cells (~10^7^) were treated with 20 nM PMA to obtain a macrophage-like cell line. Further to observe the effect of NE on the expression of C5aR, macrophages (~10^7^) were treated with purified NE (10 µg/ml) and kept at 37°C for 2 h. Simultaneously, to evaluate the effect of the inhibitor on NE, NE was pre-incubated with ecotin (1 µM) or rLdISP1/rLdISP2 (10 µM) for 30 min at 4°C. These pretreated NE was used to treat the macrophages and kept at 37°C for 2 h. The macrophages were harvested for protein isolation and the protein samples were stored at −80°C for further study.

### Semi-Quantitative PCR

UI macrophages (THP1 and hMDM) and macrophages infected with WT, ISP2OE and ISP2KD Ld parasites (for 12 h) were harvested and used for RNA isolation, using TRIzol reagent (Invitrogen) according to the manufacturer’s protocol. Simultaneously, RNA concentration was measured and cDNA was prepared by using 20 µg of RNA. cDNA was quantified and semi-quantitative PCR was performed to amplify C5aR (F-GAGGAGTACTTTCCACCAAAGG and R-AAATCGTGAGCGTGAGTAGAG). GAPDH was used as an endogenous control. The PCR was performed in thermocycler machine using thermocycling conditions -denaturation at 94°C for 5 min and 28 amplification cycles (94°C for 45 s, 56°C for 45 s, and 72°C for 1 min) followed by a final extension at 72°C for 5 min. The PCR products were run on the agarose gel (1.5%) and stained with ethidium bromide prior to analysis.

### Western blot

The protein samples were prepared from NE treated macrophages to evaluate the C5aR expression. 12% SDS-PAGE was used for the separation of protein samples. Western blot was performed by transferring the gel onto PVDF membrane, followed by blocking with 3% BSA. The expression of C5aR was analyzed by using anti-human C5aR antibody (1:1,000 dilution). Alkaline phosphatase conjugated secondary antibody (1:5,000 dilution), followed by NBT/BCIP was used to detect the band. Additionally, macrophages (THP1 and hMDM) infected with WT, ISP2KD and ISP2OE Ld parasites were harvested and used for protein isolation and Western blot analysis using antibody against the C5aR. Furthermore, Western blot was performed to observe the expression of AKT, p-AKT, PI3K, and p-PI3K proteins in UI macrophages (THP1) or wortmannin pretreated Ld infected macrophages, macrophages infected with ISP2KD, ISP2OE, and WT parasites using anti -AKT, p-AKT, PI3K, p-PI3K primary antibody, respectively. Alkaline phosphatase conjugated secondary antibody (1:5,000) followed by addition of NBT/BCIP solution was used for detection. GAPDH was taken as an endogenous control.

### Measurement of Cytokine Production

The culture supernatant of the different group of infected macrophages (THP1 and hMDM) was used to measure cytokine production of IL-12, IL-10. The cytokines level was measured by sandwich ELISA using commercially available BD OptEIA ELISA kits (BD, San Jose, CA, USA). The experiments were performed in triplicate and the data are means ± SD from three separate experiments. An asterisk (*) denotes *P* ≤ 0.05 and a double asterisk (**) denotes *P* ≤ 0.001 when compared to control.

## Results

### LdISP1 and LdISP2 Proteins Showed Strong Interaction with Classical and LP S1A Serine Peptidases (C1r, C1s, MASP1, and MASP2)

**Figure 1:**
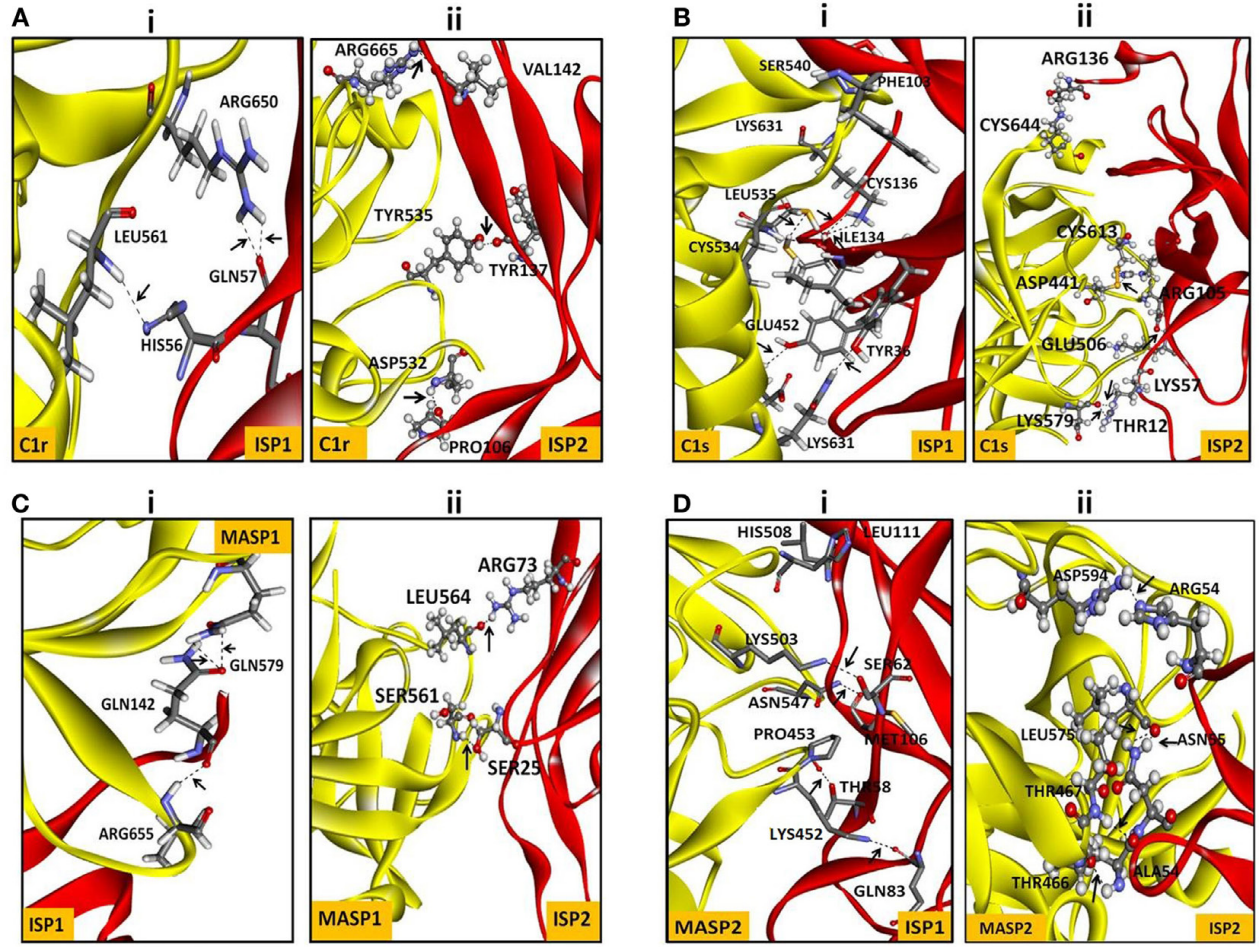
*In silico* interaction of ISP1 and ISP2 with C1r, C1s, MASP1, and MASP2. Homology model of *L. donovani* ISP1 and ISP2 proteins was obtained from our previous report (24). The interaction of ISP1 and ISP2 proteins with C1r, C1s **(A,B)** as well as with MASP1, MASP2 **(C,D)** was performed by GRAMM -X (Web Server v.1.2.0 program) software. The ISP1 interaction was shown as stick whereas ISP2 interaction was represented as ball and stick. Hydrogen bond interactions were shown as dotted black lines with an arrow mark.

Nowadays, bioinformatics play a vital role in elucidating the possible protein–protein or protein–ligand interacting residues and their mode of binding (24, 43–45). In our study, the final models of ISP1 and ISP2 was validated with Galaxy WEB server. To monitor the reliability of the model of ISP1 and ISP2, diverse statistical parameters were considered (Table 1). Significant favoured region in the Ramachandran plot was observed for both ISP1 (96.53%) and ISP2 (98.52%). Simultaneously, ERRAT gives a measure of the structural error for each residue in the protein (ISP1-83.178% and ISP2-71.875%). Precision of the model was checked using the ProSA web server by analysing Z-score for ISP1 (−3.76) and ISP2 (−4.68) respectively (Table 1). In this notion, *in silico* protein–protein interaction was performed between LdISP1/LdISP2 and the peptidase involved in classical or lectin pathway of complement channels, such as C1r, C1s, MASP1, and MASP2. Our preliminary analysis revealed that predictive model of both LdISP1 and LdISP2 formed a stable complex with C1r, C1s, MASP1, and MASP2 *via* non-covalent hydrogen bonds (H-bonds). The different interacting amino acids of protein–protein interaction have been shown in Figure 1 (Tables S1 and S2 in Supplementary Material). Notably, during interaction of C1r with ISP1/ISP2, three H-bonds were formed in both cases, i.e., between C1r–ISP1 complex and between C1r–ISP2 complex. Specifically, during C1r and ISP1 interaction the strong H-bond between C1r:ARG650:HH12 and ISP1:GLN57: O plays a crucial role in the formation of a stable ISP1–C1r complex (Figure 1Ai). During the interaction of C1s with ISP1 and ISP2, nine and eight H-bonds were formed, respectively (Figure 1Bi,ii). Moreover, CYS136 of ISP1 formed 4H-bonds with CYS534 and LYS631 of C1s (Figure 1Bi). This implies the possible central role of CYS136 of ISP1 in the formation of a stable ISP1-C1s complex. Similarly, the interface analysis also depicted that four and two H-bonds was formed during MASP1 interaction with ISP1and ISP2, respectively (Figures 1C). GLN142 of ISP1 was found to be participated most in H-bond formation (4H-bond) (Figure 1Ci). Interestingly, the formation of two strong H-bonds (distance <2Ǻ) between MASP2 and ISP1 as well as MASP2 and ISP2 implies that both ISP1 and ISP2 are capable to form a stable ISPs–MASP2 complex (Figure 1D). Additionally, the key residue, MASP2:LEU575 formed two H-bond with ISP2:ASN55 and enhances its interaction stability (Figure 1Dii). Therefore, from the *in silico* analysis, it can be speculated that both LdISP1 and LdISP2 strongly interact with the complement serine peptidases C1r, C1s MASP1, and MASP2, where H-bond profile act as one of the most driving forces for protein–protein interaction stability.

**Table 1:** Statistical data of ISP1 and ISP2 protein model.

GALAXY web	ISP1	ISP2
Poor rotamers (goal: <0.3%)	0.83%	0.00%
Favored rotamers (goal: >98%)	98.35%	98.33%
Ramachandran outliers (goal: <0.05%)	0.00%	0.00%
Ramachandran favored (goal: >98%)	96.53%	98.52%
Bad bonds: (goal: 0%)	0.00%	0.00%
ERRAT	83.178%	71.875%
Verify 3D	69.86%	88.32%
PROSA	−3.76	−4.68

**Figure 2:**
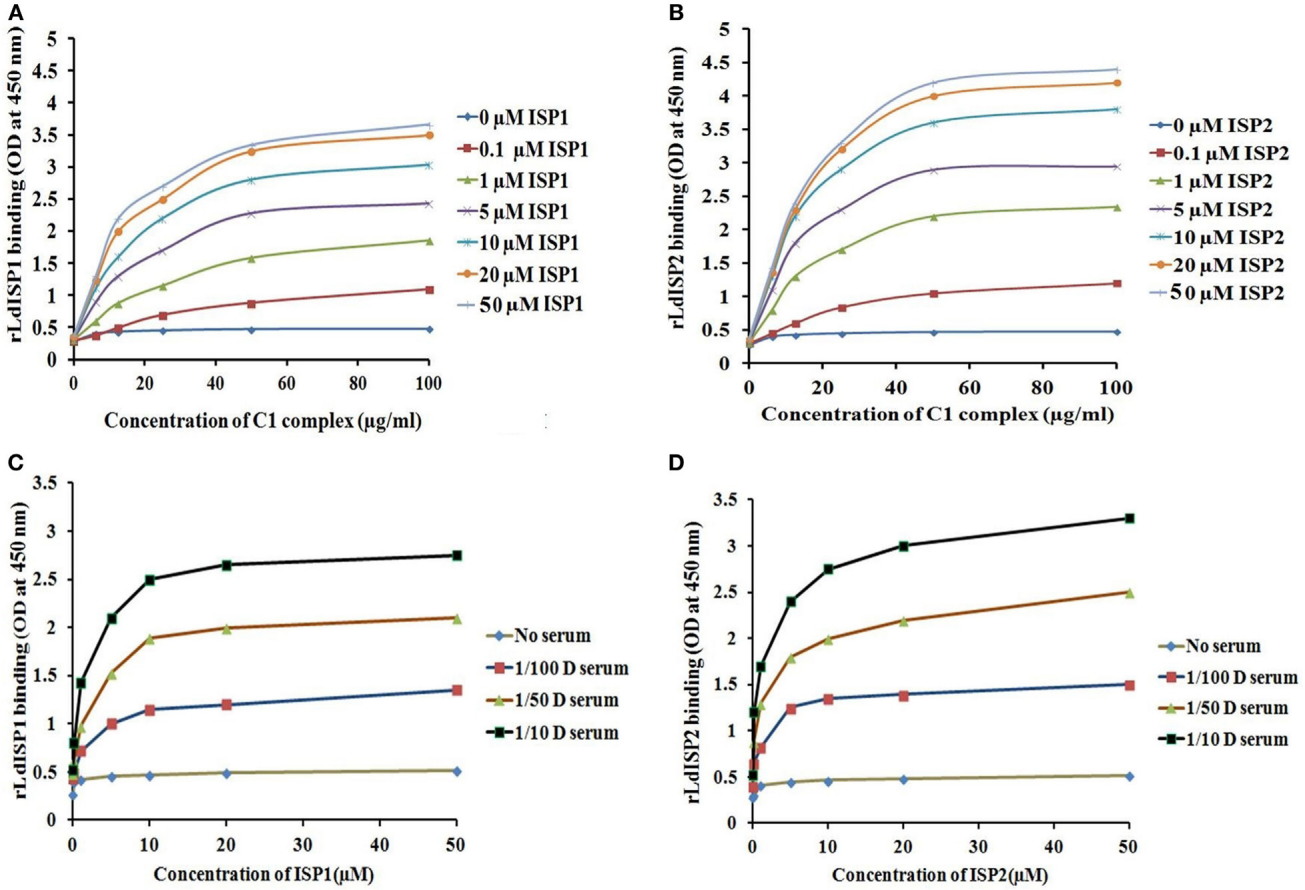
*In vitro* interaction of rLdISP1 and rLdISP2 with C1 complex and MBL-associated serine proteases (MASPs). C1 complex and normal human serum (NHS) were allowed to bound with IgM and mannan-coated plates for interaction study of C1 complex **(A,B)** and MASPs **(C,D)**, respectively. Different concentration of C1 complex (0–100 µg/ml) and different dilution of NHS (1:100, 1:50, 1:10) was used for the study. Simultaneously, different concentration of rLdISP1 and rLdISP2 (0–50 µM) was added to the C1 and MASPs bound plate and incubated at 37°C for 2 h. Bound rLdISP1 or rLdISP2 was detected with goat anti-ISP1 antibody and anti-ISP2 antibody, respectively, followed by HRP conjugated rabbit anti-goat IgG-Ab. Optical density was taken at 450 nm in ELISA plate reader. Results are representative of two independent experiments and data showed the mean of duplicate observation.

It was reported previously that the strength of the protein–protein interaction could be indirectly estimated by some measured output signals that is directly proportinal to the receptor–ligand complex formation (46). Therefore, to further assess the interaction of rLdISP1 and rLdISP2 with C1 complex and MASPs, direct binding experiment, i.e., ELISA was conducted (Figure 2). In order to quantify the interactions between the biological molecules, the determination of dissociation constants (Kd) or association constant (Ka) is one of the most important factor (46, 47). Kd represent the concentration of free ligand that is responsible for the half of the maximum saturation of receptor. In our study, Kd and Ka was determined according to Wilkinson et al. (46) during rLdISP1/rLdISP2 interaction with C1 complex (Table 2i) and MASPs (Table 2ii). At different concentration of C1 complex the dissociation constant for rLdISP1/rLdISP2 was measured and it was found inbetween the range (3.8–1.6 μM) for rLdISP1 and (3.5–1.6 µM) for rLdISP2 (Table 2i). The maximum binding affinity of rLdISP1/rLdISP2 was observed at 100 µg/ml concentration of C1 complex and assocition constant (Ka) was found to be 0.62 and 0.76 µM^−1^ for rLdISP1and rLdISP2, respectively. This result showed that low range of rLdISPs (3.8–1.6 µM) was required for half of the saturation of C1 complex. A dose-dependent saturation curve was observed by increasing concentration of rLdISP1/rLdISP2 up to 50 µM (Figures 2A,B). Similarly, a dose-dependent and saturation binding of rLdISP1/rLdISP2 was also observed with MASPs at different dilution of serum (Figures 2C,D). In different dilutions of serum, the half of the saturation was observed between 1.2–3.2 µM concentration of rLdISP1 or rLdISP2 (Table 2ii). These findings suggest that low range of rLdISP1 and rLdISP2 (1.2–3.2 µM) was required for half of the MASPs saturation. It was found that the binding capacity of rLdISPs increases with decreasing dilution of serum. Moreover, the maximum binding of rLdISPs was observed at 1:10 dilution of serum and Kd was found to be 1.4 and 1.2 µM for rLdISP1 and rLdISP2, respectively. This result showed that rLdISP2 has higher binding affinity (Ka-0.83 μM^−1^) for MASPs than rLdISP1 (Ka-0.71 μM^−1^). The control protein sample containing only C1 complex or MASPs (no ISP), taken as the negative control did not show significant optical density. These results indicated that rLdISP1 and rLdISP2 have an efficient binding affinity for both C1 complex and serum-derived MASPs.

**Table 2:** Binding affinity of LdISP1 and *L. donovani* inhibitor of serine peptidases 2 (LdISP2) with (i) C1 complex and (ii) serum MBL-associated serine proteases.

(i)				

Concentration of C1 complex used	LdISP1 (Kd) (µM)	LdISP2 (Kd) (µM)	LdISP1 (Ka) µM^-1^	LdISP2 (Ka) µM^-1^
6.25 µg/ml	3.8	3.5	0.26	0.28
12.5 µg/ml	3.5	3.2	0.28	0.31
25 µg/ml	2.8	3	0.33	0.35
50 µg/ml	2.2	2.0	0.45	0.5
100 µg/ml	1.6	1.3	0.625	0.76

**(ii)**				

**Dilution (D) of human serum used**														
1/100 D	3.2	3.0	0.31	0.33
1/50 D	2.2	1.9	0.45	0.52
1/10 D	1.4	1.2	0.71	0.83

### LdISP2 Inhibits MASP2 Enzyme Activity

**Figure 3:**
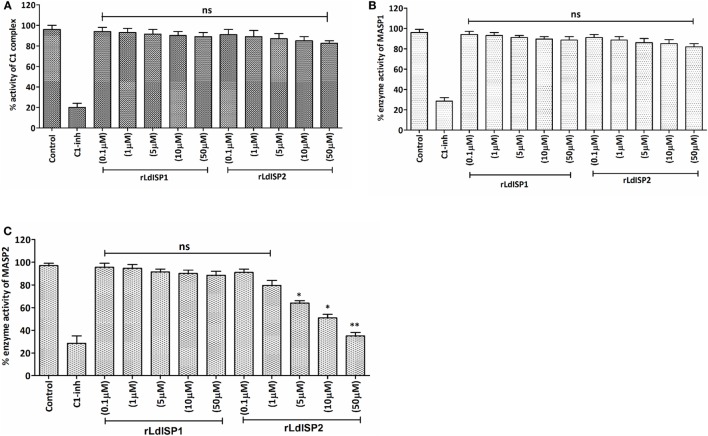
Inhibitory effects of rLdISP1 and rLdISP2 on C1 complex and MBL-associated serine proteases (MASPs). The inhibitory effect of rLdISP1 and rLdISP2 on C1 complex **(A)**, MASP1 **(B)**, and MASP2 **(C)** was monitored by enzymatic assay. Microtitration plates were coated either with human IgM or mannan. Simultaneously, C1 complex and normal human serum (as a source of MASPs) was added in the antibody and mannan bound plate, respectively. The plates were incubated with rLdISP1/rLdISP2 (0.1–50 µM) or C1-inhibitor or without inhibitor for 1hr. The C1 complex or mannan-coated plates without inhibitor were used as a control. Relative enzyme activity of C1 complex and MASP2 **(A,C)** was analysed by subsequent addition of chromogenic substrate, i.e., N-α-Cbz-l-lysine thiobenzyl ester (Z-l-Lys-SBzl) and DTNB [5,5-di thio-bis-(2 nitro-benzoic acid)] after incubation at 37°C for 1 h. The absorbance was recorded at 405 nm for every 10 min for 1 h. Simultaneously, for MASP1 activity, **(B)** the chromogenic substrate, i.e., VPR–7-amino-4 methyl coumarin (AMC) was added to the MASPs bound plate and incubated for 1 h at 37°C. The cleaved substrate was monitored by spectrofluorometer using an excitation wavelength of 355 nm and an emission wavelength of 450 nm. The experiments were performed in triplicate and the data were expressed as means ± SD from three independent experiments. Kruskal–Wallis with Dunn’s multiple comparison test was used to evaluate statistical significance for comparing the data of different groups. An asterisk (*) denotes *P* ≤ 0.05, double asterisk (**) denotes *P* ≤ 0.001 and “ns” denotes non-significant as compared to control.

In order to investigate the effect of rLdISP1 and rLdISP2 on C1 complex and MASP1/MASP2 activity, specific enzyme assay was carried out (Figure 3). No significant inhibition of the enzyme activity of C1 complex and MASP1 was observed in presence of rLdISP1 and rLdISP2 (concentration up to 50 µM) (Figures 3A,B) when compared to control. However, the enzyme activity of serum-derived MASP2 was reduced by ~30, ~44, and ~60% in the presence of 5, 10, and 50 µM of rLdISP2, respectively, when compared to the untreated serum (control) (Figure 3C). C1-inh (C1-inh), significantly inhibited the proteolytic activity of both enzyme C1 complex and MASP1/MASP2.

### LdISP2 Inhibit MASP2 Dependent C4 Cleavage

**Figure 4:**
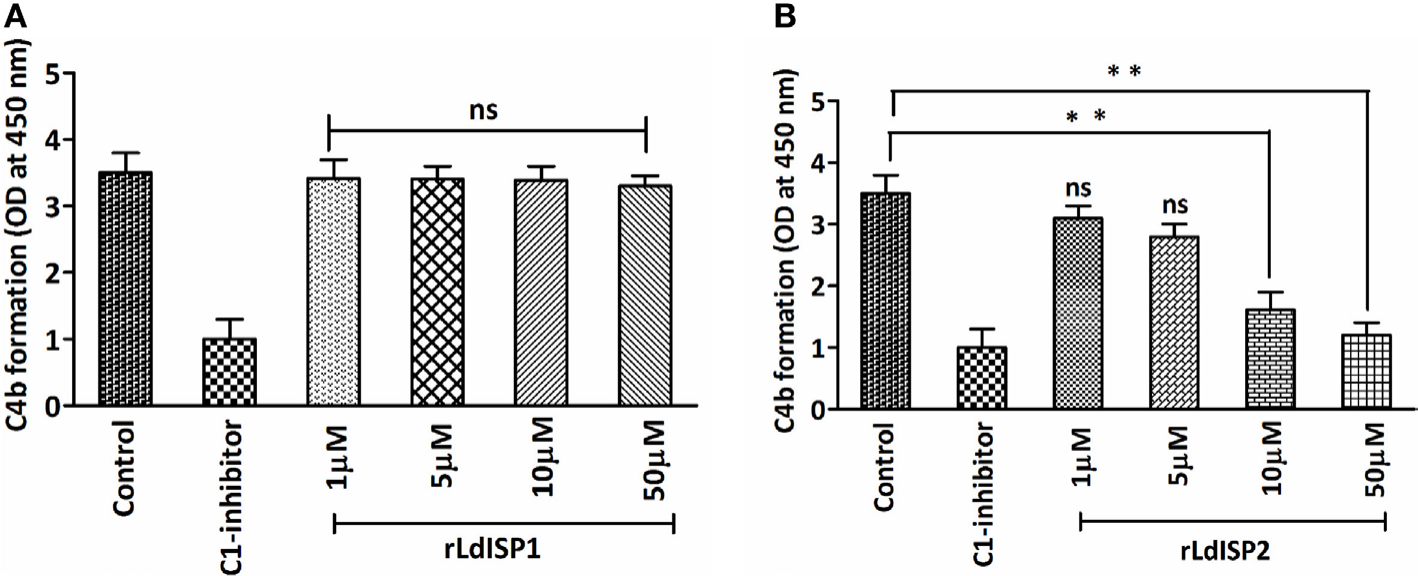
*L. donovani* Inhibitor of Serine Peptidases 2 (LdISP2) inhibits lectin pathway mediated C4b formation. Inhibitory effect of rLdISP1 **(A)** and rLdISP2 **(B)** on MBL-associated serine proteases (MASPs) was assessed by sandwich ELISA. Mannan-coated microtitration plate was incubated with normal human serum (1/10 v/v), NHS pretreated with C1-inhibitor and rLdISP1/rLdISP2 (0–50 µM). The ELISA plate bound with serum-derived MASPs was used as a control. Purified C4 molecule (0.2 µg/ml) was added to the plate and incubated for 2 h at 37°C. C4 cleavage was monitored by the relative formation of C4b molecule after addition of C4b antibody followed by HRP-conjugated secondary antibody. The absorbance was measured at 450 nm. The experiments were performed in triplicate and data are means ± SD from three separate experiments. Kruskal–Wallis with Dunn’s multiple comparison tests was used for comparing the data of different groups to evaluate statistical significance. Asterisk (*) denotes *P* ≤ 0.05, double asterisk (**) denotes *P* ≤ 0.001 and “ns” denotes non-significant as compared to control.

It was previously reported that MASP2 has the ability to cleave C4 molecule to produce C4a and C4b (4). Therefore, in this study, we aimed to investigate the effect of rLdISP1 and rLdISP2 on the MASPs-mediated cleavage of C4 molecule that might affect C4b formation. The ELISA plate coated with serum-derived MASPs was used as a control. No significant inhibition in C4b formation was observed in presence rLdISP1 even at higher concentration (50 µM), when compared to control (Figure 4A). However, a significant inhibition in C4b formation was observed in presence of rLdISP2 and C1-inh. C4b formation was found to be decreased by ~2.1 and ~2.9-fold in presence of 10 and 50 µM rLdISP2, respectively, when compared to control (Figure 4B). Since we have observed previously that rLdISP2 was unable to inhibit the enzyme activity of MASP1, it is evident that the reduction in C4 cleavage occurred solely due to reduced activity of MASP2 by rLdISP2.

### LdISP2 Reduces Lectin Pathway Associated C3c and C5c Formation

**Figure 5:**
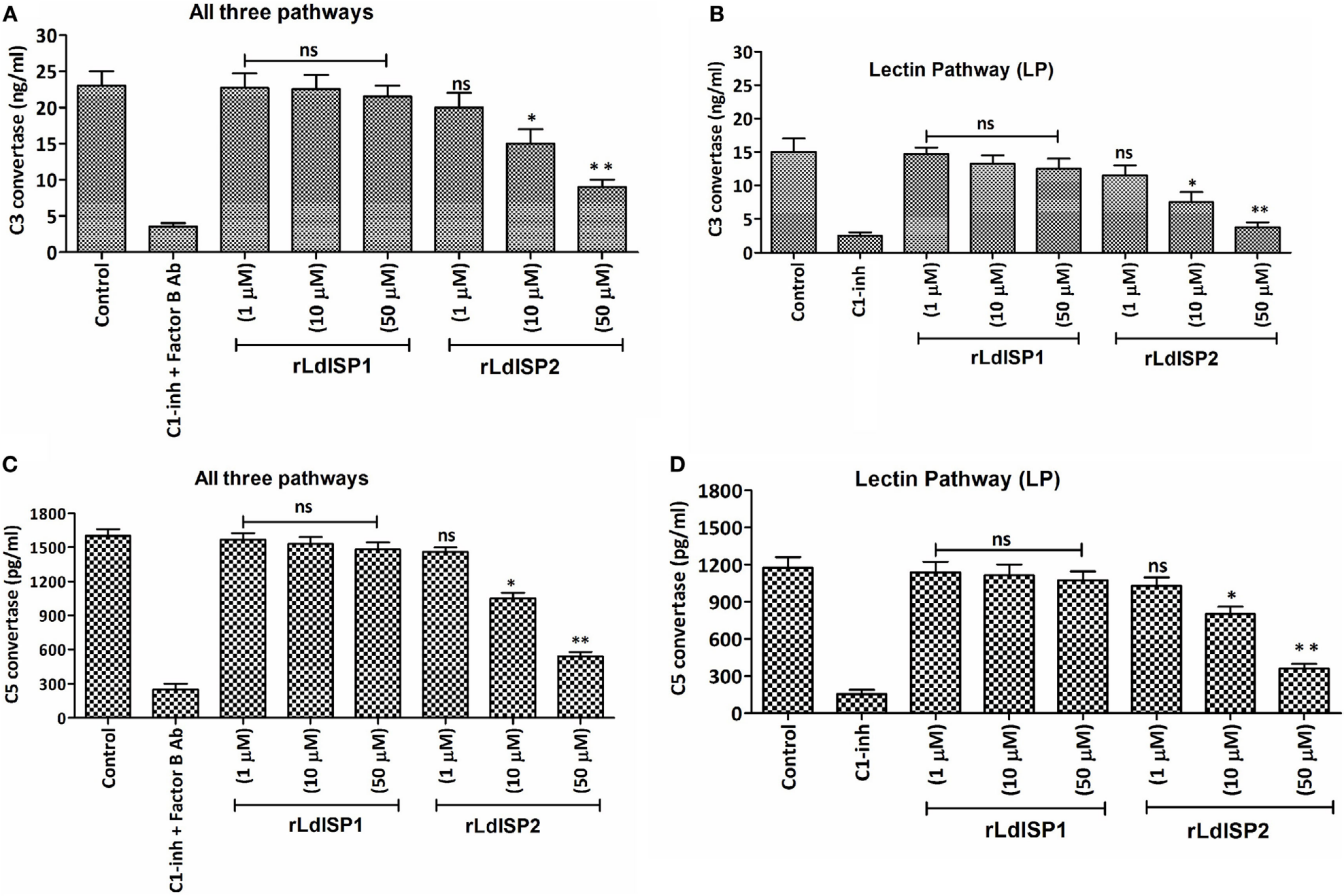
Effect of LdISPs on the formation of C3 convertase (C3c) and C5 convertase (C5c). The activity of C3c and C5c was measured to monitor the formation of C3c and C5c *via* all complement pathways or specifically *via* lectin pathway in presence or absence of rLdISP1and rLdISP2. Normal human serum or lectin pathway-specific serum was incubated with different concentration (0–50 µM) of rLdISP1 and rLdISP2, C1-inhibitor (C1-inh) or C1-inh + factor B Ab at 37°C for 2 h. *Leishmania* promastigotes (~2 × 10^6^) were added to the different group of treated serum and incubated at 30°C with shaking at 300 rpm for 5–10 min. Formation of C3c **(A,B)** and C5c **(C,D)** were accessed by sandwich ELISA. The experiments were performed in triplicate and data are means ± SD from three separate experiments. An asterisk (*) denotes *P* ≤ 0.05, double asterisk (**) denotes *P* ≤ 0.001 and “ns” denotes non-significant as compared to control, Kruskal–Wallis test with Dunn’s multiple comparison tests.

Addition of *Leishmania* parasites to the active human serum lead to activate complement pathways resulting in the formation of C3 and C5c. The effect of the rLdISPs on C3 and C5c formation was assessed by using human serum or human serum pre-incubated with different concentration (0–50 µM) of rLdISP1 or rLdISP2. C3c and C5c formation *via* all complement pathways or individually *via* lectin pathway were accessed by sandwich ELISA. ~1.5- and ~2.5-fold reduction in C3c by all pathways was observed in presence of 10 and 50 µM of rLdISP2, respectively, when compared to control (Figure 5A). Simultaneously, by lectin pathway a ~2- and ~4-fold reduction in C3c was observed in presence of 10 and 50 µM of rLdISP2, respectively, when compared to control (Figure 5B). Similarly, the C5c formation by all three pathways was reduced by ~1.5- and ~2.9-fold at 10 and 50 µM rLdISP2, respectively, when compared to control (Figure 5C). Simultaneously, a ~1.4- and ~3.2-fold reduction in C5c by lectin pathway was observed at 10 and 50 µM of rLdISP2, respectively, when compared to control (Figure 5D). Serum pre-incubated with “C1-inh + Factor B antibody” and C1-inh was used as a negative control that significantly reduced the convertase formation by all contributing pathways and lectin pathway, respectively. No significant change in C3c, C5c formation was observed in presence of rLdISP1 when compared to the untreated NHS or LPSS (control).

### LdISP2 Inhibits MAC Formation and Complement-Mediated Lysis *via* Lectin Pathway

**Figure 6:**
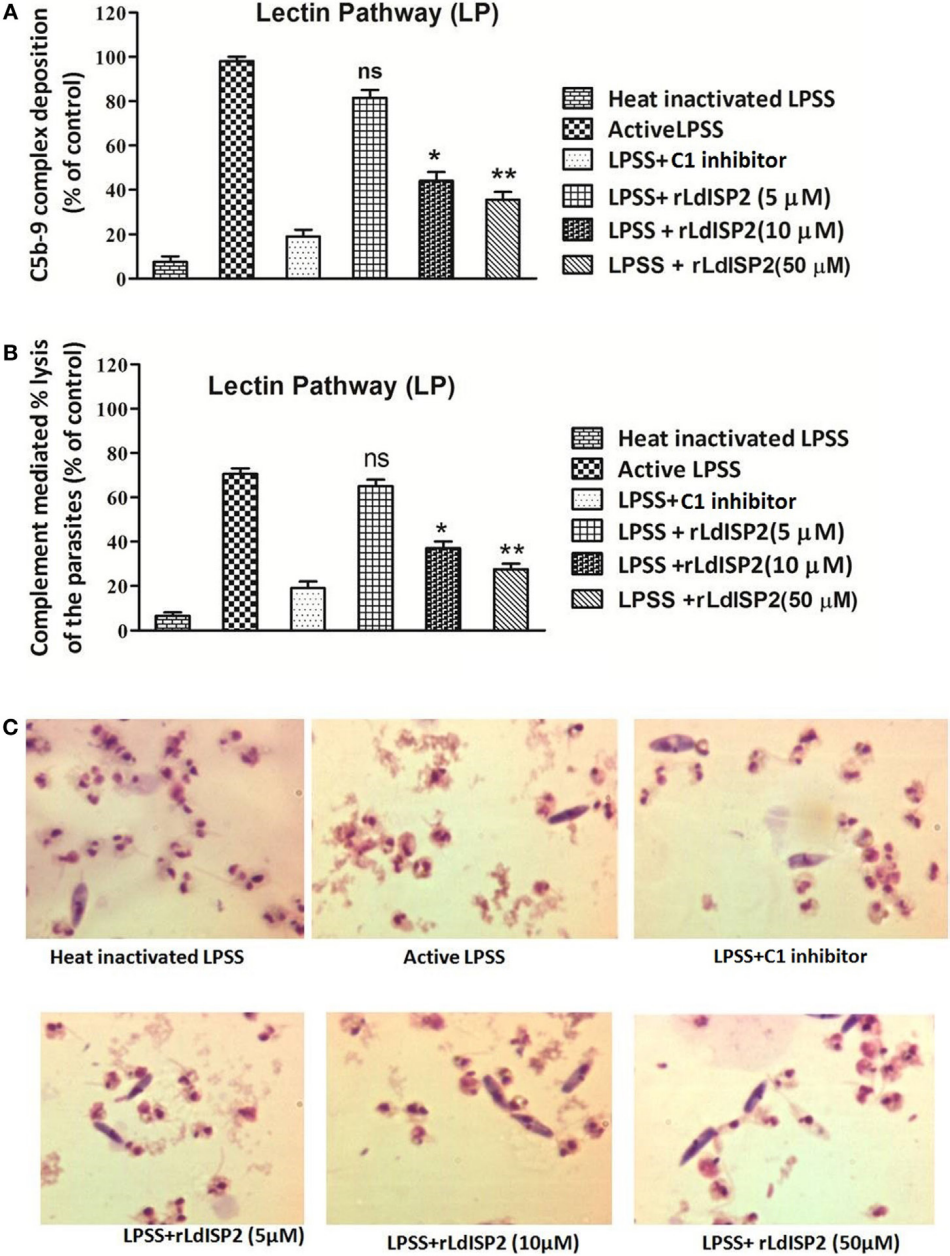
Effect of rLdISP2 on membrane attacking complex (MAC) formation and complement-mediated lysis. Lectin pathway (LP) was activated by using lectin pathway specific serum (LPSS). MAC (C5b-9 complex) formation and subsequent parasite lysis was monitored in presence or absence of rLdISP2. *Leishmania* promastigotes (~1 × 10^7^) were either treated with heat-inactivated LPSS, active LPSS and LPSS pretreated with C1-inhibitor (10 nM) or rLdISP2 (5–50 µM). The relative formation of C5b-9 complex **(A)** and complement-mediated lysis (*via* LP) **(B)** was measured by ELISA. LP mediated killing of *Leishmania* parasites was analysed in presence of rLdISP2. The lysis of the parasites was visualized by optical microscopy **(C)**. The experiments were performed in triplicate and data are means ± SD from three separate experiments. An asterisk * denotes *P* ≤ 0.05 and double asterisk ** denotes *P* ≤ 0.001 when compared to control (Student’s *t*-test).

Altered level of C3c and C5c in presence of rLdISP2 would ultimately lead to affect C3b formation, MAC (C5b-9 complex) formation and pathogen killing by complement-mediated lysis. Therefore, C3b deposition and MAC formation individually by the CP, LP, AP was analyzed in case of *Leishmania* donovani infection, and it was observed that the lectin pathway is the most activated pathway among all the three pathways (CP, LP, AP) (SI). As previous findings suggested that LdISP2 inhibits LP associated MASP2; therefore, MAC formation and complement-mediated lysis by LP was analyzed in presence of rLdISP2. ~1.5- and ~2.3-fold decrease in MAC formation was observed *via* LP in presence of 10 and 50 µM of rLdISP2 when compared to active LPSS (control) (Figure 6A). Simultaneously, a ~1.8- and ~2.5-fold reduction in the parasites lysis *via* LP was also observed in presence of 10 and 50 µM of rLdISP2, respectively, when compared to control (Figure 6B). Microscopic observation of the parasite also revealed that the parasite lysis *via* lectin pathway was significantly reduced in presence of rLdISP2 (5–50 µM) (Figure 6C). However, no significant changes in MAC formation and parasite lysis was observed in presence of heat inactive serum and C1-inh treated serum.

### LdISP2 Induced the Expression of C5aR by Inhibiting NE Activity

**Figure 7:**
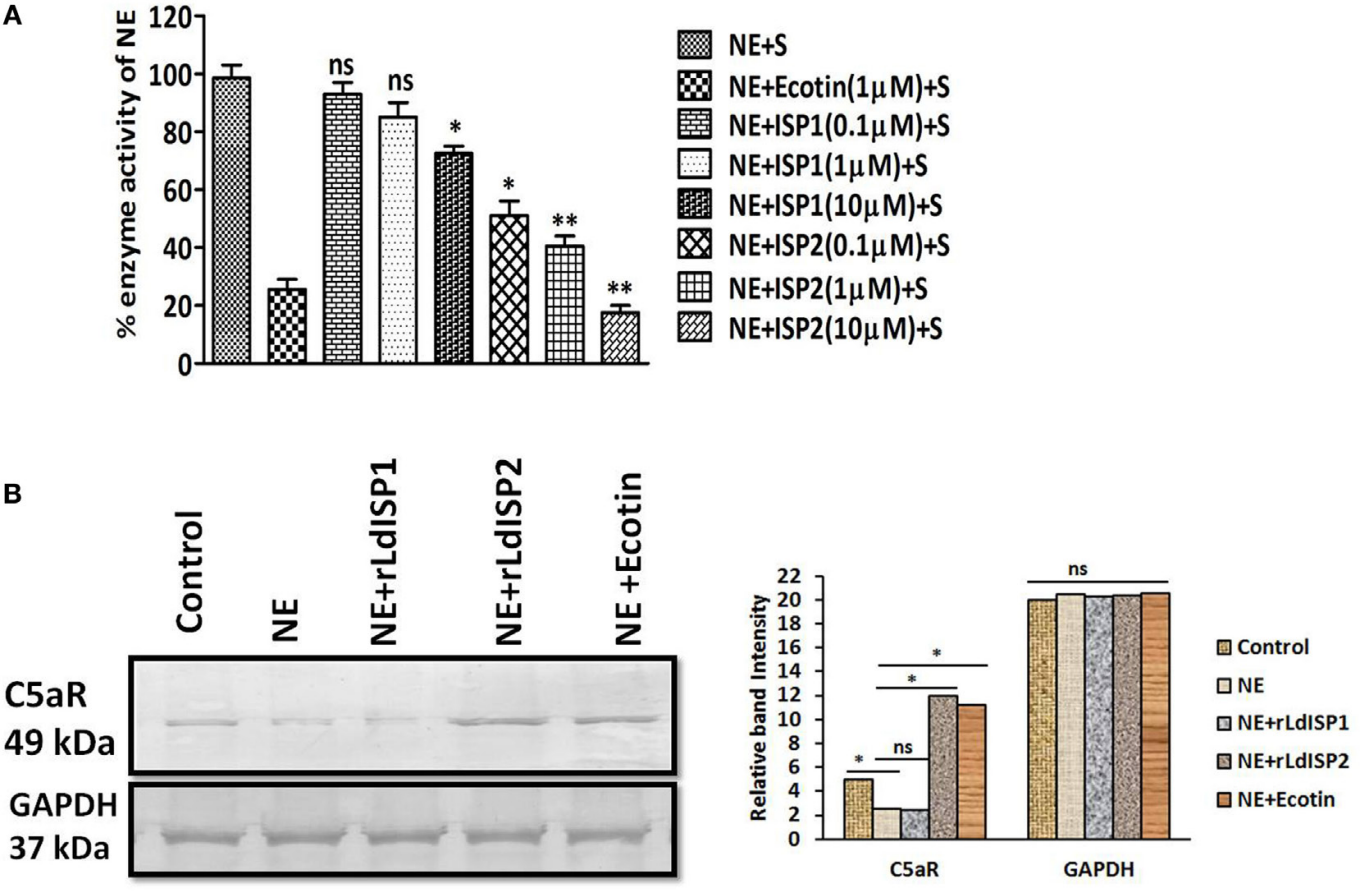
Effect of LdISPs on neutrophil elastase (NE) and C5aR expression. The inhibitory effect of rLdISPs (rLdISP1 and rLdISP2) on NE was accessed by enzymatic assay **(A)**. NE was either left untreated or treated with ecotin (1 µM) or rLdISP1/rLdISP2 (0.1–10 µM) and incubated on ice for 30 min followed by subsequent addition of chromogenic substrate. Enzymatic hydrolysis of the substrate was monitored by measuring the release of fluorescence (excitation 380 nm, emission at 460 nm) in the spectrofluorometer. **(B)** Macrophages were either left untreated or treated with NE (10 µg/ml), or NE pre-incubated with an inhibitor such as ecotin (1 µM) or rLdISP1/rLdISP2 (10 µM) followed by incubation for 2 h at 37°C. The expression of C5aR was analysed by western blot. GAPDH was used as an endogenous control. Densitometric analysis was done to monitor the relative band intensity. The experiments were performed in triplicate and data are means ± SD from three separate experiments. The blot image is representative of a single experiment. Kruskal–Wallis test with Dunn’s multiple comparisons was performed. An asterisk (*) denotes *P* ≤ 0.05 and a double asterisk (**) denotes *P* ≤ 0.001 when compared to control.

The activity of NE in presence of rLdISP1or rLdISP2 was analyzed by enzymatic assay using chromogenic substrate. ~49, ~60, and ~82% reductions in enzyme activity was observed in presence of 0.1, 1, and 10 µM of rLdISP2, respectively (Figure 7A). However, only ~27% inhibition of enzyme activity was observed in presence of rLdISP1 (10 µM). Additionally, the effect of NE and NE specific inhibitor including rLdISP1/rLdISP2 on the expression of macrophage C5aR was analysed (Figure 7B). An approximately 1.6-fold decrease in C5aR expression was observed in NE (10 µg/ml) treated macrophages. Furthermore, a ~2.5-fold increase in C5aR expression was observed in macrophages treated with NE pretreated with rLdISP2 (10 µM), when compared to control. In presence of NE pretreated with ecotin (1 µM) the C5aR expression was increased by ~2-fold when compared to control. However, no significant change in expression of C5aR was observed in macrophages treated with NE pre-incubated with rLdISP1 when compared to control. Our results demonstrated that NE activity leads to decrease in C5aR expression whereas inhibition of NE activity by ecotin or rLdISP2 significantly induces the expression of C5aR.

### LdISP2 Increases the Expression Level of C5aR in Macrophages

**Figure 8:**
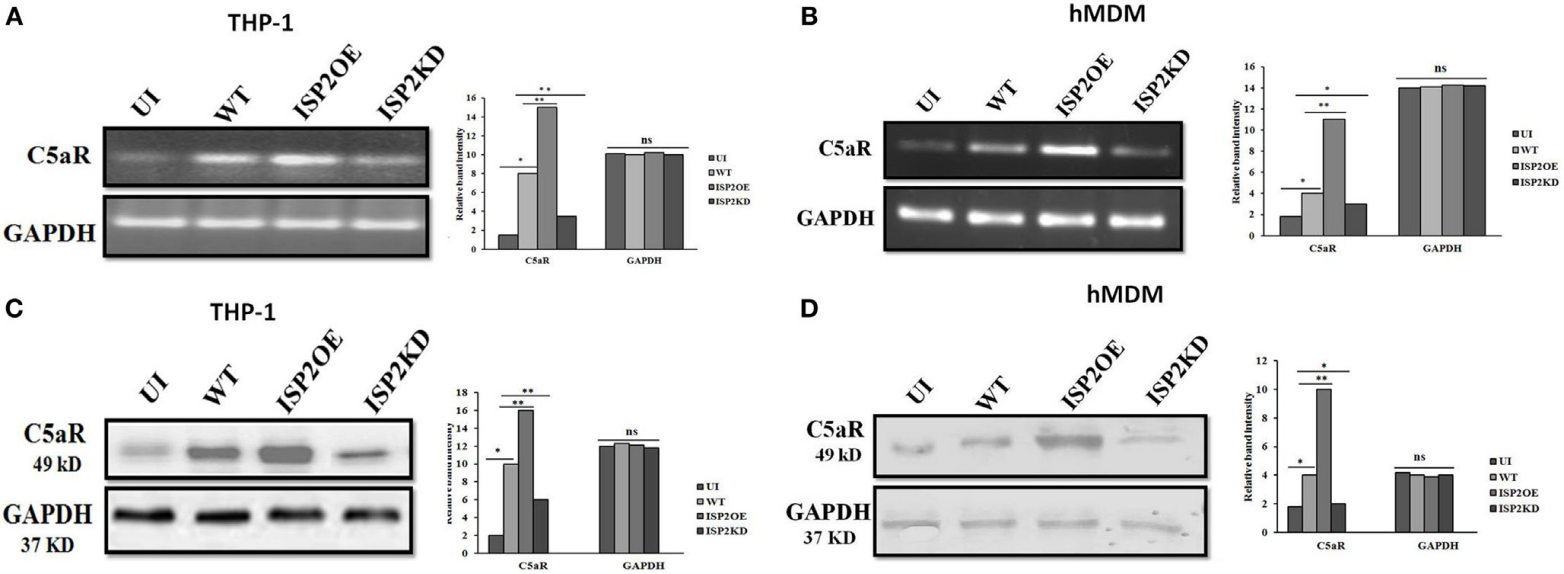
Effect of *Leishmania donovani* inhibitor of serine peptidases 2 on C5aR expression in infected macrophages. THP1 monocyte-derived macrophages **(A,C)** and human monocyte-derived macrophages (hMDM) **(B,D)** were either left UI or infected with WT, ISP2OE and ISP2KD *Leishmania* parasites for 12 h. The expression level of C5aR was analysed at transcript level by semi-quantitative PCR **(A,B)** and at protein level by western blot **(C,D)**. Densitometric analysis showed the relative fold increase in the band intensities of C5aR expressed in the different group of infected parasites when compared to the UI or WT infected macrophages. Experiments were performed in triplicate. Gel and blot images are representative of a single experiment. An asterisk (*) denotes *P* ≤ 0.05 and the double asterisk (**) denotes *P* ≤ 0.001, when compared to UI or WT infected as applicable, “ns” denotes non-significant changes (Mann–Whitney *U* test). Abbreviations: UI, uninfected; WT, wild-type; ISP2OE, ISP2 overexpressed; ISP2KD, ISP2 knocked down.

The expression of C5aR was analyzed at the transcript and protein level in macrophages (THP1 and hMDM) infected with WT, ISP2OE, and ISP2KD Ld parasites. Interestingly, at transcript level in case of THP1 cells, a ~5-fold increase in C5aR expression was observed in WT infected macrophages when compared to UI macrophages. However, a ~1.8-fold increase and a ~2.3-fold decrease in expression of C5aR was observed in ISP2OE and ISP2KD infected macrophages, respectively, when compared to WT infected macrophages (Figure 8A). However, in hMDM cells, a significant ~2.2-fold increase in C5aR expression was observed in WT infected macrophages when compared to UI macrophages whereas, a significant ~2.7-fold increase and a ~1.3-fold decrease in expression of C5aR was observed in ISP2OE and ISP2KD infected macrophages, respectively, when compared to WT infected macrophages (Figure 8B).

Simultaneously, at protein level in THP1 cells, a ~5-fold increase in the expression of C5aR was also observed in WT infected macrophages when compared to UI macrophages. Moreover, a ~1.6-fold increase in expression of C5aR in ISP2OE infected macrophages and a ~1.7-fold decrease in C5aR expression in ISP2KD infected macrophages was observed when compared to WT Ld infected macrophages (Figure 8C). Similarly, in case of hMDM cells, the expression of C5aR was found to be significantly increased by ~2.2-fold in WT infected macrophages when compared to UI macrophages. Simultaneously, we have observed a significant ~2.5-fold increase in expression of C5aR in ISP2OE infected macrophages and a ~2-fold decrease in C5aR expression in ISP2KD infected macrophages when compared to WT Ld infected macrophages (Figure 8D). All these results demonstrated that knocked down of LdISP2 significantly downregulate the expression of host macrophages C5aR expression.

### LdISP2 Is Involved in Activation of PI3K/AKT Pathway to Promote Parasite Survival

**Figure 9:**
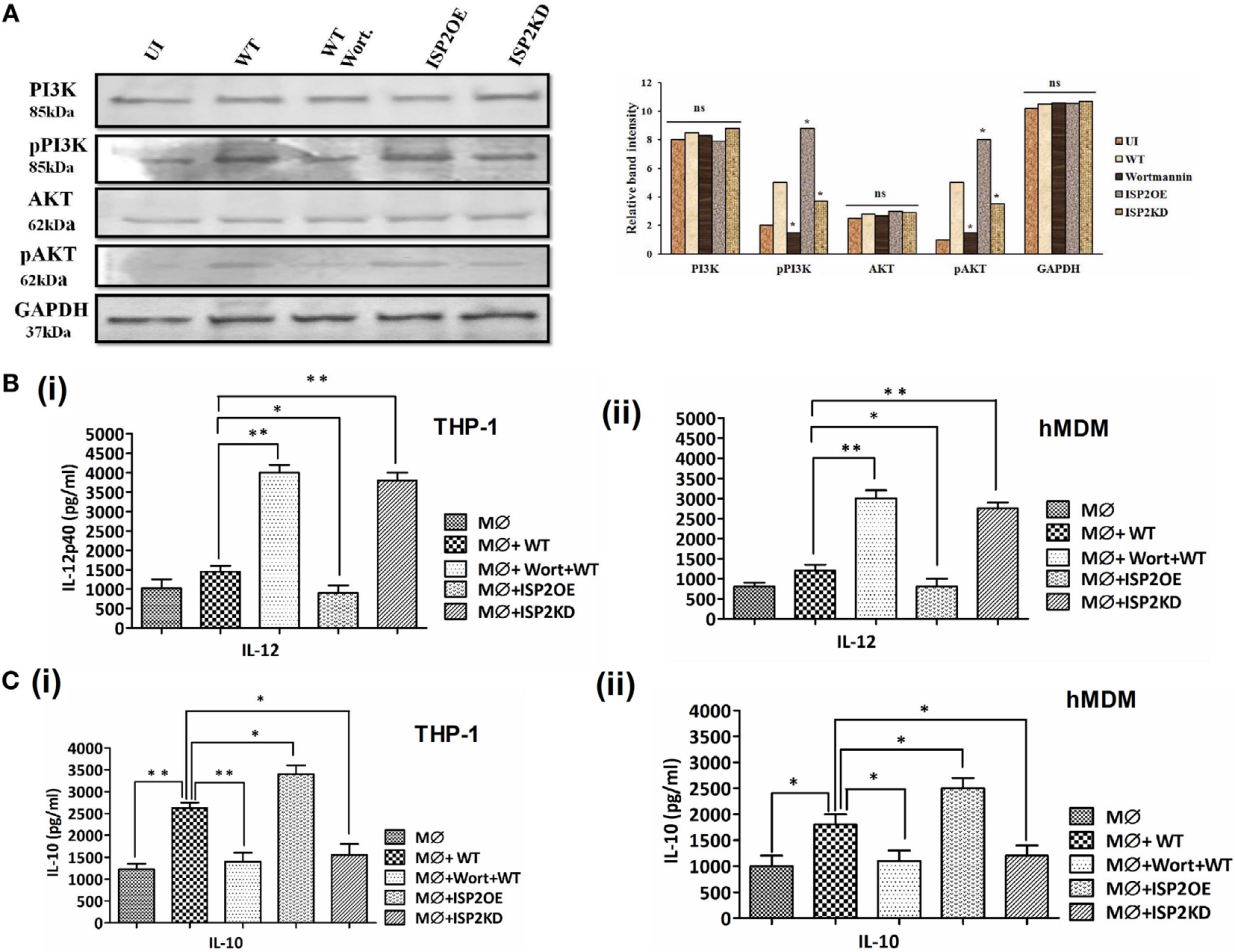
Effect of LdISP2 on activation of PI3K/AKT pathway. Macrophages (THP1 or hMDM) were either left untreated or pretreated with wortmannin followed by infection by wild-type (WT), ISP2 overexpressed (ISP2OE) and ISP2KD *Leishmania* parasites for 12 h. The expression of PI3K, p-PI3K, AKT, and p-AKT was evaluated in THP1 cells at the protein level by western blot **(A)**. The release level of IL-12 **(B)**, and IL-10 **(C)** in the culture supernatant of THP1 monocyte-derived macrophages **(B,C)** (i) and human monocyte-derived macrophages (hMDM) **(B,C)** (ii) was measured by sandwich ELISA. Blot image is representative of a single experiment. The experiments were performed in triplicate and the data are means ± SD from three separate experiments. An asterisk (*) denotes *P* ≤ 0.05, when compared to uninfected or WT infected as applicable. Evaluation of the statistical significance of the data was performed by Kruskal–Wallis with Dunn’s multiple comparison tests.

The expression of PI3K, p-PI3K, AKT and p-AKT was analyzed in case of WT, ISP2KD, and ISP2OE *Leishmania* infection to macrophages (THP1 cells) (Figure 9A). Interestingly, a ~2- and ~1.5-fold increase in phosphorylation of PI3K and AKT was observed in WT infected macrophages when compared to UI. However, a ~3.3- and ~3.2-fold decrease in p-PI3K and p-AKT expression was observed in wortmannin-treated macrophages infected with WT Ld when compared to untreated macrophages infected with WT Ld. Additionally, a ~2-fold increase in p-PI3K and ~1.6-fold increase p-AKT expression was observed in macrophage infected with ISP2OE parasites when compared to WT infected macrophages. Moreover, a ~2- and ~1.6-fold decrease in expression of p-PI3K and p-AKT were observed, respectively, in macrophages infected with ISP2KD parasites when compared to WT Ld-infected macrophages. These results demonstrated that *Leishmania* infection induces PI3K and AKT activation and their phosphorylation is significantly increased in the presence of LdISP2.

Simultaneously, the involvement of PI3K and AKT in Th1/Th2 balance was further assessed by measuring the cytokines, i.e., IL-12 and IL-10 at the released level in macrophages (THP1 and hMDM) infected with *Leishmania* parasites (Figures 9B,C). In case of THP1 cells, in wortmannin pretreated macrophages infected with WT parasites, IL-12 level increased ~2.7-fold. Additionally, a ~1.6-fold decrease and ~2.6-fold increase in IL-12 was observed in macrophages infected with ISP2OE and ISP2KD parasites, respectively (Figure 9Bi). Similarly, in case of hMDM cells, in wortmannin pretreated macrophages infected with WT parasites, IL-12 level increased ~2.5-fold. However, a ~2.3-fold decrease and ~1.5-fold increase in IL-12 was observed in macrophages infected with ISP2OE and ISP2KD parasites, respectively (Figure 9Bii). Moreover, the IL-10 level was also found to be significantly altered in macrophages (THP1 and hMDM) infected with ISP2KD and ISP2OE parasites. Interestingly, in THP1 cells we observed a ~2-fold increase in IL-10 in WT infected macrophages when compared to noninfected macrophages and a ~1.8-fold decrease in IL-10 level in wortmannin pretreated WT infected macrophages when compared to untreated macrophages infected with WT Ld. We have also found significant ~1.6-fold decrease and ~1.3-fold increase in IL-10 production in macrophages infected with ISP2KD and ISP2OE parasites, respectively (Figure 9Ci). Similarly, in case of hMDM cells we have found a ~1.8-fold increase in IL-10 in WT infected macrophages when compared to noninfected macrophages and a ~1.6-fold decrease in IL-10 level in wortmannin pretreated WT infected macrophages when compared to untreated macrophages infected with WT Ld. Simultaneously, we have found significant ~1.5-fold decrease and ~1.4-fold increase in IL-10 production in macrophages infected with ISP2KD and ISP2OE parasites, respectively (Figure 9Cii).

**Figure 10:**
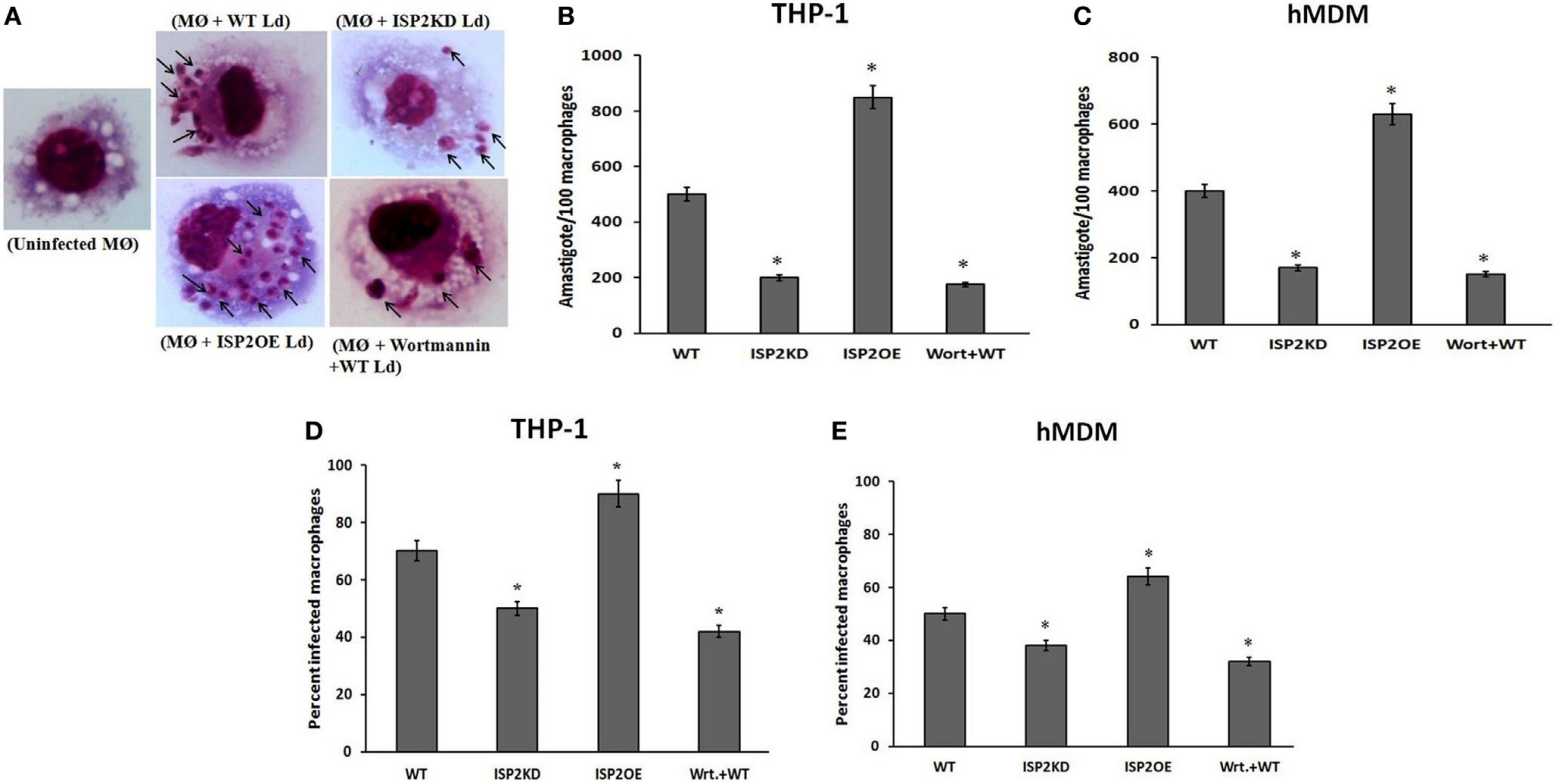
Effect of LdISP2 on survival of ***Leishmania donovani*** inside macrophages. THP1 monocyte-derived macrophages and human monocyte-derived macrophages (hMDM) were either left untreated or pretreated with wortmannin followed by infection with WT, ISP2KD and ISP2OE *Leishmania* parasites for 24 h. After infection intracellular parasites inside the macrophages (THP1) were visualized by staining the cells with Giemsa followed by optical microscopy at 100× oil immersion **(A)**. The parasite load inside THP1 cells **(B)** and hMDM cells **(C)** was measured by counting the number of intracellular amastigotes per 100 macrophages. The rate of infection was analyzed by counting the percent infected macrophages [THP1 and hMDM **(D,E)**]. Each determination was made in triplicate and the values were expressed as mean ± SD for three independent experiments. A Student’s *t*-test was used to evaluate statistical significance; Asterisk (*) denotes *P* < 0.05 whereas, “ns” denotes non-significant change. Abbreviations: WT, wild-type; Wrt + WT, Wortmannin-treated macrophages infected with wild type; ISP2OE, ISP2 overexpressed; ISP2KD, ISP2 knocked down; MØ + WT Ld, Macrophages infected with WT Ld; MØ + ISP2KD Ld, Macrophages infected with ISP2 knocked down Ld; MØ + ISP2OE-Ld, Macrophages infected with ISP2 overexpressed Ld.

To evaluate the effect of LdISP2 on the survival of *Leishmania* inside macrophages (THP1 and hMDM), parasite load and percentage of infectivity were measured in wortmannin pretreated macrophages infected with WT Ld and macrophages infected with WT, ISP2KD, ISP2OE *Leishmania* parasites for 24 h. Microscopic picture after Giemsa staining displaying different parasite load inside infected macrophages (THP1) (Figure 10A). In case of THP1 cells, an approximately ~2.5-fold decrease and a ~1.7-fold increase in parasite load were observed in macrophages infected with ISP2KD and ISP2OE Ld parasites, respectively, when compared macrophages infected with WT parasites (Figure 10B). Parasite load was found to be decreased by ~2.8-fold in wortmannin pretreated infected macrophages when compared to WT Ld infected macrophages (Figure 10B). Moreover, in case of hMDM cells, the parasite load was found to be ~2.3-fold decreased and ~1.75-fold increased in macrophages infected with ISP2KD and ISP2OE Ld parasites, respectively, when compared macrophages infected with WT parasites. Parasite load was severely affected in wortmannin pretreated infected macrophages and found to be ~2.6-fold decreased when compared to WT Ld infected macrophages (Figure 10C). Simultaneously, the percentage of infectivity was also severely affected in macrophages (THP1 and hMDM) infected with ISP2KD and ISPEOE parasites respectively when compared WT infected macrophages. In case of THP1 cells the percentage of infectivity was reduced by ~1.6-fold in wortmannin pretreated Ld infected macrophages when compared to WT Ld infected macrophages. The percentage of infectivity, in case of macrophages infected with ISP2KD parasites was ~1.4-fold less than WT Ld infected macrophages whereas, in case of ISP2OE Ld infected macrophages the percent infectivity was found to be ~1.3-fold higher than the WT Ld infected macrophages (Figure 10D). Moreover, in case of hMDM cells, the percentage of infectivity was decreased by ~1.6-fold in wortmannin pretreated Ld infected macrophages when compared to WT Ld infected macrophages. However, the percentage of infectivity was reduced by ~1.3-fold and increased by ~1.3-fold in case of macrophages infected with ISP2KD and ISP2OE Ld, respectively, when compared to WT Ld infected macrophages (Figure 10E). Therefore, our data demonstrated that LdISP2 activates PI3K/AKT pathway and increased IL-10 level that promotes parasite survival inside the host, whereas downregulation of ISP2 led to decreased activation of PI3K/AKT pathway and increased IL-12 level that severely affects parasite survival inside the host.

## Discussion

During transmission of *Leishmania* from the sand fly to the mammalian host, the parasites are deposited in host skin through the regurgitation of promastigote secretory gel and migrate to the host blood stream (48). Inside blood stream *Leishmania* encounter complement attack in form of innate immunity (49). Out of three complement activation route, lectin pathway has a crucial role in host defense against protozoan parasites and it is the most contributing pathway in parasite clearance during *Leishmania* infection (11). This pathway has a major role during early infection, whereas the CP is activated after the host develops a specific antibody response (50).

The previous study suggested that trypanosomes and *Leishmania* encode various proteins and develop several mechanisms to overcome the complement effects during their infection that help the parasites to survive inside the host (13, 17, 18, 51–56). Complement network is a hub of S1A SPs (7) that may be targeted by parasite-derived inhibitor of serine peptidases (LdISP2), contributing a major role in parasite survival against complement attack. Moreover, it has also been reported that L. major ISP2 has a role in inhibition of N.E, promoting parasite survival inside the macrophages (22). Since NE downregulate the expression level of C5aR (23), therefore it could be possible that LdISP2 might also influence C5aR mediated host PI3K/AKT pathway for parasite survival. In the present study, we showed the engagement of LdISP2 in relation to host response.

An *in silico* interaction, study was performed to see the protein-protein interaction between catalytic SP domain (trypsin fold) of C1r, C1s, MASP1, MASP2 and LdISPs (LdISP1/LdISP2). These complement proteins belong to S1A family and bear the catalytic domains having trypsin or chymotrypsin-like activity. According to Perona et al. (57), the structure of trypsin (S1 site) revealed that its catalytic site consists of Serine-195, His-57, and Asp-102 with several other key amino acids, such as Tyr-172, Ser-190, Asp-189, and Gly-216, which determine the substrate specificity and responsible for the enzyme efficiency. Therefore, we have analyzed the amino acids of LdISP1/LdISP2 that interacts with the catalytic residue of the SP domain of S1A proteases. Our interface analysis depicted that several H-bonds are formed between the catalytic residue of SP and LdISPs during the interaction, that inferred a strong interaction between LdISPs (LdISP1 and LdISP2) and S1A peptidases (C1r, C1s, MASP1, MASP2) of the complement system and these interactions could be effective in functional prospects.

We have further validated our *in silico* protein–protein interaction data by *in vitro* methods. ELISA has been used previously to assess the interaction of CRT of *Trypanosoma cruzi* with C1q protein of human (14). It is the indirect method to analyse the protein–protein interaction. In the present study, ELISA was also performed to examine the interaction of rLdISP1 and rLdISP2 with selected S1A peptidases (C1 complex and MASPs). During protein–protein (receptor–ligand) interaction, the binding parameter could be determined by the measurement of output signal such as absorbance and it could be assumed that this output signal is directly proportional to the protein–ligand complex formation. Association constant (Ka) represents the binding affinity between two proteins and represent the strength of the reverse interaction between two or more than two proteins (46, 47). On the other hand dissociation constant (Kd), a reciprocal of Ka (1/Ka) is also a quantitative indicator of the stability of receptor–ligand complex formation. A plot of fractional saturation of receptor verses free ligand determines the dissociation constant (Kd) of free proteins (46, 58). Lower the value of Kd represent higher stability of the receptor–ligand complex formation, whereas higher value of Kd represent less stable complex formation. In our study strong saturation binding was observed between rLdISPs and C1 complex/MASPs. Both C1 complex and MASPs showed significant binding affinity with rLdISP1 and rLdISP2. However, rLdISP2 showed stronger affinity with C1 complex and MASPs when compared to rLdISP1. Our data suggested that both rLdISPs strongly bind with C1 complex and MASPs, which supports the *in silico* findings.

Functional consequences of the interaction of rLdISP2 with C1 complex or MASPs was further assessed by enzymatic assay. The interaction and inhibition studies suggested that although, the strong interaction exists between rLdISP1/rLdISP2 and C1 complex but this interaction did not affect the functional activity of the C1 complex. Furthermore, the interaction of rLdISP2 with MASPs showed a significant inhibition in enzyme activity of serum-derived MASPs with MASP2 compared to MASP1. These evidences suggested that rLdISP2 inhibits MASP2 enzyme activity. The inhibitory property of rLdISP2 might be due to the presence of inhibition site, consisting of P1 reactive methionine that was found to be absent in LdISP1 (21, 24). Our findings are in agreement with the previous report of Gaboriaud et al. (59), where they have shown that *Escherichia coli* derived ecotin (ISP like) bound to the SP domain of MASP2 but not with C1r, C1s. Therefore, our findings evidence that LdISP2 inhibits MASP2 activity but not MASP1.

Previous findings by Ambrus et al. (60) and Megyeri et al. (61) have demonstrated that MASP1 cannot cleave C4 (60, 61), whereas MASP2 has the ability to cleave C4 to produce C4b molecule. The production of C4b results in C3c formation that contributes a major role in lectin pathway (LP) activation (10). We hypothesized that inhibition of the MASP2 by LdISPs might results in downregulation of the activation of the LP. In our study, C4 cleavage assay was performed in presence or absence of rLdISPs to monitor C4b production, using serum-derived MASPs. Interestingly, C4b production gets reduced in presence of rLdISP2, the reduction in C4b production occurred due to the inhibitory effect of rLdISP2 on MASP2, as our previous finding suggested that rLdISP2 has the inhibitory effect on MASP2 rather than MASP1.

Since C3 and C5c formation are central stages of complement cascade, the presence of complement inhibitors might attenuates convertase formation or accelerates the dissociation of convertase complexes (41). It was reported that complement C2 receptor inhibitor trispanning of *T. cruzi* inhibits the formation of the C3c by interacting with the complement component C2 (51). Additionally, leishmanial GP63, a major surface glycoprotein interacts with C3b results in its conversion to inactive C3b and interferes with C3c formation (17). In our study, the presence of rLdISP2 might also affect the down-stream convertase formation of LP. In the present study, we have also analyzed the formation of C3 and C5c in the presence or absence of rLdISP1/rLdISP2. Our study showed that C3 and C5c formation is impaired in presence of rLdISP2 that might be due to inhibition of MASP2 activity that affects the convertase formation *via* LP. Therefore, our study indicates that ISP2 of *L. donovani* prevents C3 and C5c formation which would lead to reduce parasite lysis by complement attack.

It has been reported that serum exposure to the *L. donovani* (62), *Leishmania major* and *Leishmania mexicana* (63) promastigotes leads to activate the LP *via* binding of serum mannose-binding protein to the major surface glycoconjugates of the parasites and play important role in complement-mediated lysis. In contrary, the lysis of the parasites gets significantly reduced by the use of human serum depleted of lectin molecules MBL and ficolins (11). The functional activation of complement pathway could be observed by the measurement of C3b molecule and C5b-9 (MAC) deposition on the parasite or the microorganism surface (42, 53, 55). Therefore, in our study, the comparative analysis of the activation of different complement pathways by *L. donovani* parasites was assessed by measurement of C3b deposition and immune complex (C5b-9) formation. We have found that lectin pathway is the most activated pathway by *L. donovani* among all the pathways. Furthermore, we have also perceived that MAC formation and lysis of the parasites get significantly reduced in presence of rLdISP2. The possible reason might be that the presence of LdISP2 impaired the function of MASP2 that affects MAC formation, resulting in reduction in parasite lysis. Therefore, our study clearly indicates that *Leishmania* parasites escape from lysis mediated by lectin pathway using ISP2. A similar study was also performed by Ferreira et al. (13), where they have shown that *T. cruzi* CRT bind to the mannan-binding lectin of the human complement system and inhibit the lectin pathway. Another report by Punetes et al. (53) suggested that *L. major* evade the host complement-mediated lysis by a modified LPG on promastigote surface that prevent the insertion of MAC into parasite membrane.

Additionally, in our study, we have also tried to decipher the role of ISP2 in the late stage of infection. Therefore, in the current study, we used ISP2KD and ISP2OE parasite to investigate the molecular pathway through which these inhibitors exert their role in *Leishmania*-macrophage interaction. Previously Faria et al. (22) reported that ISP2 has a role in parasite survival inside macrophages by inhibiting NE-mediated TLR-4 activation. Based on it, we decided to investigate the involvement of LdISP2 in NE-mediated C5aR signaling. Importantly, we observed that ISP2 inhibits NE activity as suggested by Faria et al. (22). In addition, reduced expression of C5aR was observed in presence of NE, whereas increased expression of C5aR was observed in presence of NE treated with rLdISP2. From these findings, it is evident that presence of NE downregulates C5aR expression and significant inhibition of NE activity by LdISP2 results in upregulation of C5aR expression. Our finding was supported by the previous report by Van den Berg et al. (23) where they showed that the presence of NE cleave C5a receptor and downregulate its expression.

It was reported that during *Leishmania* infection, activation of C5aR occurs, which in turn activates PI3K pathway resulting in downregulation of IL-12 (64). In the current study, we observed a significant increase in the expression of C5aR in macrophages (THP1 cells and hMDM) infected with WT parasites when compared to noninfected macrophages. We have observed a significant decrease in expression of C5aR both at the transcript and protein level in macrophages infected with ISP2KD parasites, when compared to WT Ld infected macrophages. The possible explanation behind our finding might be that C5aR expression is affected by reduced enzyme activity of NE due to the presence of LdISP2 that leads to increased expression of C5aR.

According to Hawlisch et al. (65, 66) C5aR signaling has a significant role in promoting *Leishmania* infection and progression of the disease by negative regulation of TLR-4-induced immune responses (65, 66). Besides that Strainic et al. (67) suggested that C5aR mediate their effects *via* PI-3-kinase-γ-dependent AKT phosphorylation, providing a crosslink between G-protein coupled receptor signaling and cytokine production. Therefore, any impairment in C5aR expression severely affects the signaling cascade through it and thereby affects parasite survival. In this study, the phosphorylation of PI3K/AKT was found to be increased in *L. donovani* infected macrophages when compared to noninfected macrophages. Our findings demonstrated that *Leishmania* infection induces PI3K/AKT pathway which was agreement with the previous reports by Ruhland et al. (68) and Kima et al. (69). The involvement of LdISP2 in C5aR-mediated signaling was evaluated by measuring the phosphorylation status of p-AKT and p-PI3K during macrophage infection with ISP2KD and ISP2OE parasites. A significant reduction in phosphorylation of PI3K and AKT was observed in macrophages infected with ISP2KD parasites whereas increased phosphorylation was observed in macrophages infected with ISP2OE parasites when compared to WT Ld infected macrophages. The plausible reason might be that the reduction in C5aR expression reduces the signaling through G protein receptor that led to the reduction in the phosphorylation status of both PI3K and AKT molecules in macrophages infected with ISP2KD parasites.

IL-12 is a key pro-inflammatory cytokine, produced mainly by the phagocytic cells and acts as an effective stimulant for the induction of IFN-γ mediated Th1 response during visceral leishmaniasis (VL) that help in parasite clearance (70). The persistence of the parasites and disease progression is favored by a major immunosuppressive cytokine IL-10 that promotes Th2 mediated response (71). In our study, we observed that IL-12 level significantly increased in Ld infected macrophages pretreated with wortmannin when compared to untreated infected macrophages. From these findings, it is evident that PI3K/AKT pathway is involved in the IL-12 production. Additionally, a significant increase in IL-12 was observed in macrophages infected with ISP2KD parasites whereas IL-12 level was found to be significantly decreased in macrophages infected with ISP2OE parasites. As the previous study reported that C5aR mediated signaling is involved in the induction of PI3K signaling which in turn suppress the production of IL-12 (72); therefore, based on our findings, we could say that overexpression of ISP2 induces the signaling through C5aR, which in turn downregulate the level of IL-12. Simultaneously, a significant increase in IL-10 was also observed in WT Ld infected macrophages, whereas significant decreased level of IL-10 was observed in wortmannin pretreated Ld infected macrophages when compared to UI macrophages. In addition, a significant decrease in IL-10 level was found in macrophages infected with ISP2KD parasites whereas a significant increase in IL-10 was observed in macrophages infected with ISP2OE parasites when compared to the macrophages infected with WT Ld parasites. Our results demonstrated that ISP2 mediated engagement of C5aR and PI3K/AKT pathway differentially altered the level of IL-12 and IL-10. From our study, it is evident that downregulation of LdISP2 impaired the C5aR mediated Th1/Th2 balance that might be responsible for *L. donovani* survival inside the host. Therefore, we have analyzed the killing of ISP2KD and ISP2OE parasites inside the macrophages to see the crucial role of ISP2 in *Leishmania* survival. We observed that the survival of ISP2KD parasites severely reduced at 24 h after infection whereas the survival of ISP2OE parasites gets enhanced when compared to WT parasites. All these findings indicates that presence of LdISP2 exert a beneficial effect for intracellular *L. donovani* survival inside macrophages due to the inhibition of PI3-Kinase regulated IL-12 production, whereas downregulation of ISP2 enhances the parasite killing.

**Figure 11:**
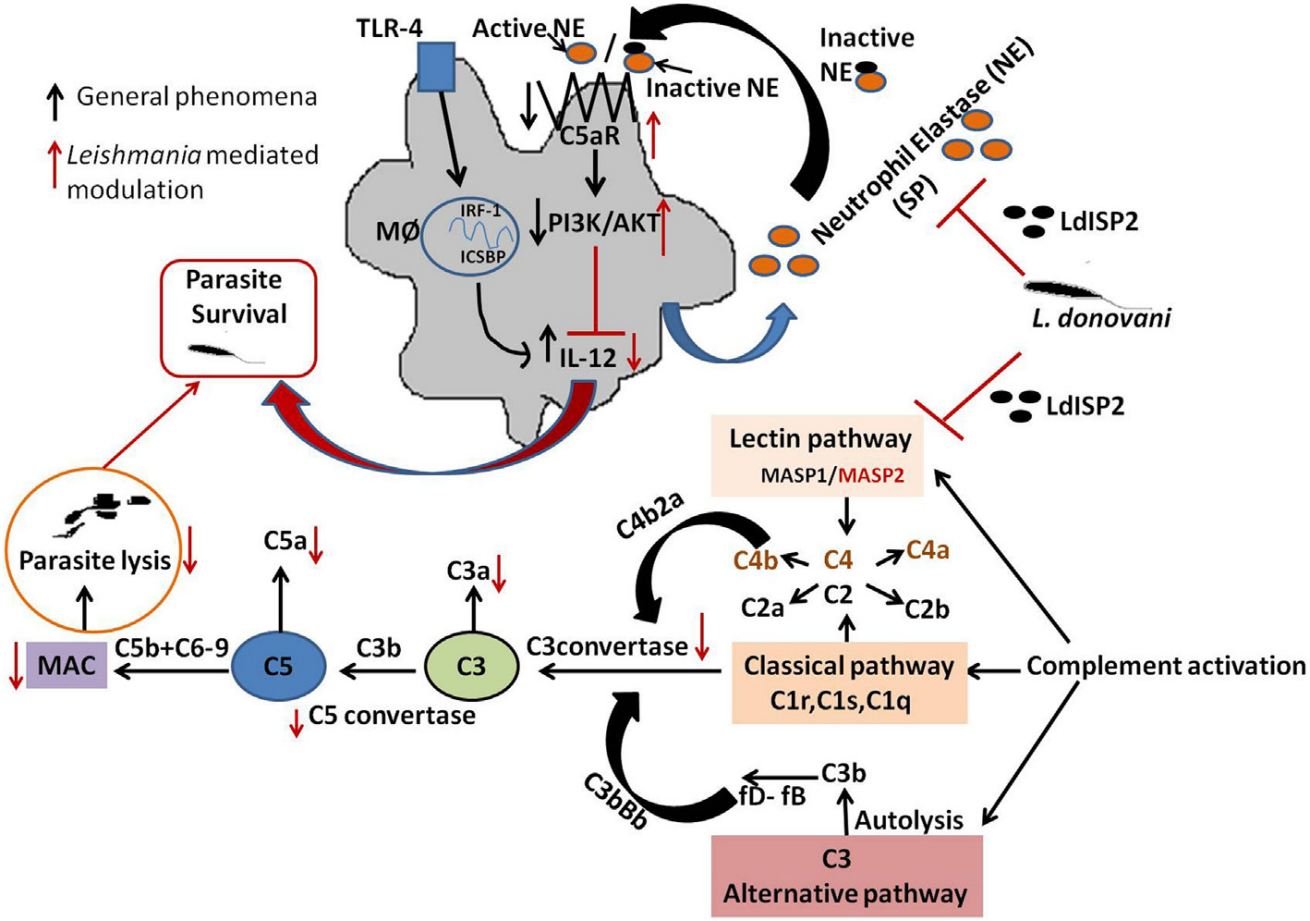
Schematic representations demonstrating the role LdISP2 in the modulation of host response. The model illustrates that the survival of *Leishmania donovani* parasites inside host depends on their capacity to resist the complement attack and to modulate the host immune response by using LdISP2. In one way, *Leishmania* ISP2 inhibit the activation of lectin pathway by interaction with MASP2. Inhibition of MASP2 by LdISP2 results in less production of C3 and C5 convertase leading to decreased membrane attacking complex (MAC) formation. Reduced MAC formation decreases complement-mediated lysis of the parasites. In this way, the *Leishmania* parasites defend itself from complement-mediated attack by using LdISP2. On another way, LdISP2 inhibits utrophil elastase (NE) activity and upregulate C5aR expression. Induced expression of C5aR activates the PI3K/AKT pathway. Phosphorylation of PI3K/AKT decreases IL-12 level. *L. donovani* utilises theses two different strategies for its own survival by using LdISP2. The black arrow indicates the normal phenomena and the red arrow indicates the phenomena modulated by *L. donovani*.

Understanding the host–pathogen interaction might open a new window in the development of new therapeutics against infectious diseases. The current knowledge of the complement system highlights the importance of LdISP2 as a key molecule against the host complement attack. Our findings indicate that LdISP2 has the significant inhibitory effect on MASP2, affecting the activation of lectin pathway *via* impairment of C3 and C5c formation and leading to decrease in parasite lysis *via* reduced formation of MAC (Figure 11). On the other hand, downregulation of LdISP2 significantly reduces the expression of C5aR and C5aR mediated PI3K/AKT signaling that affects the survival of the parasites by altering the Th1/Th2 cytokines levels (Figure 11). In conclusion, we proposed that *Leishmania* derived ISP2 protect the parasite from complement-mediated lysis by inhibiting host lectin pathway and activating PI3K/AKT pathway. Therefore ISP2 of *L. donovani* can be targeted for effective drug development against VL.

## Ethics Statement

In this study, the human serum was collected from peripheral blood of healthy volunteers after written informed consent that had been obtained with the specific permit by the ethical committee of Rajendra Memorial Research Institute of Medical Sciences (ICMR), Agamkuan, Patna, India. The institutional human ethical committee of RMRIMS (Patna, India), has approved all the protocol used in this study (Reference ID-04/IEC/2014).

## Author Contributions

The experiment was designed by SV, AM, SD, and PD. The experiments were performed by SV, AM, and MYA. Data were analysed by SV, AM, and PD. The reagents, materials and analysis tools were provided by MYA, AjayK, KA, AKG, AK, VK, and PD. The paper was written by SV, AM, and PD.

## Conflict of Interest Statement

The authors declare that the research was conducted in the absence of any commercial or financial relationships that could be construed as potential conflicts of interest.

## References

[1] WHO. (2011). Available from: http://www.whoint/en/

[2] Santos-Mateus D Passero F Rodrigues A Valério-Bolas A Silva-Pedrosa R Pereira M The battle between *Leishmania* and the host immune system at a glance. Int Trends Immun (2016) 4:2326–3121. 10.18281/iti.2016.2.3

[3] Gupta G Oghumu S Satoskar AR. Mechanisms of immune evasion in leishmaniasis. Adv Appl Microbiol (2013) 82:155. 10.1016/B978-0-12-407679-2.00005-3 23415155 PMC3697132

[4] Cestari I Evans-Osses I Schlapbach LJ de Messias-Reason I Ramirez MI. Mechanisms of complement lectin pathway activation and resistance by trypanosomatid parasites. Mol Immunol (2013) 53(4):328–34. 10.1016/j.molimm.2012.08.015 23063472

[5] Lambris JD Ricklin D Geisbrecht BV. Complement evasion by human pathogens. Nat Rev Microbiol (2008) 6(2):132. 10.1038/nrmicro1824 18197169 PMC2814840

[6] Gal P Barna L Kocsis A Zavodszky P. Serine proteases of the classical and lectin pathways: similarities and differences. Immunobiology (2007) 212(4):267–77. 10.1016/j.imbio.2006.11.002 17544812

[7] Yousef GM Elliott MB Kopolovic AD Serry E Diamandis EP. Sequence and evolutionary analysis of the human trypsin subfamily of serine peptidases. Biochim Biophys Acta (2004) 1698(1):77–86. 10.1016/j.bbapap.2003.10.008 15063317

[8] Sato T Endo Y Matsushita M Fujita T. Molecular characterization of a novel serine protease involved in activation of the complement system by mannose-binding protein. Int Immunol (1994) 6(4):665–9. 10.1093/intimm/6.4.665 8018603

[9] Goldsbay RA Kindt TJ Barbara AO Kuby J The complement system in Immunology, 5th ed. (2003). p. 301–18.

[10] Wagner E Frank MM. Therapeutic potential of complement modulation. Nat Rev Drug Discov (2010) 9(1):43. 10.1038/nrd3011 19960015

[11] Cestari IDS Krarup A Sim RB Inal JM Ramirez MI. Role of early lectin pathway activation in the complement-mediated killing of *Trypanosoma cruzi*. Mol Immunol (2009) 47(2):426–37. 10.1016/j.molimm.2009.08.030 19783051

[12] Cestari I Ramirez MI. Inefficient complement system clearance of *Trypanosoma cruzi* metacyclic trypomastigotes enables resistant strains to invade eukaryotic cells. PLoS One (2010) 5(3):e9721. 10.1371/journal.pone.0009721 20300530 PMC2838796

[13] Ferreira V Valck C Sánchez G Gingras A Tzima S Molina MC The classical activation pathway of the human complement system is specifically inhibited by calreticulin from *Trypanosoma cruzi*. J Immunol (2004) 172(5):3042–50. 10.4049/jimmunol.172.5.3042 14978109

[14] Ramírez G Valck C Molina MC Ribeiro CH López N Sánchez G. *Trypanosoma cruzi* calreticulin: a novel virulence factor that binds complement C1 on the parasite surface and promotes infectivity. Immunobiology (2011) 216(1):265–73. 10.1016/j.imbio.2010.04.001 20472323

[15] Valck C Ramírez G López N Ribeiro CH Maldonado I Sánchez G Molecular mechanisms involved in the inactivation of the first component of human complement by *Trypanosoma cruzi* calreticulin. Mol Immunol (2010) 47(7):1516–21. 10.1016/j.molimm.2010.01.019 20153898

[16] Fischer E Ouaissi MA Velge P Cornette J Kazatchkine MD. gp 58/68, a parasite component that contributes to the escape of the trypomastigote form of *T. cruzi* from damage by the human alternative complement pathway. Immunology (1988) 65(2):299. 2973433 PMC1384928

[17] Brittingham A Morrison CJ McMaster WR McGwire BS Chang KP Mosser DM. Role of the *Leishmania* surface protease gp63 in complement fixation, cell adhesion, and resistance to complement-mediated lysis. J Immunol (1995) 155(6):3102–11. 7673725

[18] Spath GF Garraway LA Turco SJ Beverley SM The role(s) of lipophos-phoglycan (LPG) in the establishment of *Leishmania major* infections in mammalian hosts. Proc Natl Acad Sci U S A (2003) 100(16):9536–41. 10.1073/pnas.1530604100 12869694 PMC170953

[19] Alam MN Das P De T Chakraborti T. Identification and characterization of a *Leishmania donovani* serine protease inhibitor: possible role in regulation of host serine proteases. Life Sci (2016) 144:218–25. 10.1016/j.lfs.2015.12.004 26656469

[20] Lima APCA Mottram JC Trypanosomatid-encoded inhibitors of peptidases: unique structural features and possible roles as virulence factors. Open Parasitol J (2010) 4:132–8. 10.2174/1874421401004010132

[21] Eschenlauer SC Faria MS Morrison LS Bland N Ribeiro-Gomes FL DosReis GA Influence of parasite encoded inhibitors of serine peptidases in early infection of macrophages with *Leishmania major*. Cell Microbiol (2009) 11(1):106–20. 10.1111/j.1462-5822.2008.01243.x 19016791 PMC2659362

[22] Faria MS Reis FC Azevedo-Pereira RL Morrison LS Mottram JC Lima APC. *Leishmania* inhibitor of serine peptidase 2 prevents TLR4 activation by neutrophil elastase promoting parasite survival in murine macrophages. J Immunol (2011) 186(1):411–22. 10.4049/jimmunol.1002175 21098233 PMC3119636

[23] Van den Berg CW Tambourgi DV Clark HW Hoong SJ Spiller OB McGreal EP. Mechanism of neutrophil dysfunction: neutrophil serine proteases cleave and inactivate the C5a receptor. J Immunol (2014) 192:1787–95. 10.4049/jimmunol.1301920 24446515

[24] Verma S Das S Mandal A Ansari MY Kumari S Mansuri R Role of inhibitors of serine peptidases in protecting *Leishmania donovani* against the hydrolytic peptidases of sand fly midgut. Parasit Vectors (2017) 10(1):303. 10.1186/s13071-017-2239-9 28645315 PMC5481909

[25] Mandal A Das S Kumar A Roy S Verma S Ghosh AK l-Arginine uptake by cationic amino acid transporter promotes intra-macrophage survival of *Leishmania donovani* by enhancing arginase-mediated polyamine synthesis. Front Immunol (2017) 8:839. 10.3389/fimmu.2017.00839 28798743 PMC5526900

[26] Singh AK Pandey RK Siqueira-Neto JL Kwon Y-J Freitas-Junior LH Shaha C Proteomic-based approach to gain insight into reprogramming of THP-1 cells exposed to *Leishmania donovani* over an early temporal window. Infect Immun (2015) 83:1853–68. 10.1128/IAI.02833-14 25690103 PMC4399049

[27] Ko J Park H Heo L Seok C GalaxyWEB server for protein structure prediction and refinement. Nucleic Acids Res (2012) 40:W294–7. 10.1093/nar/gks493 22649060 PMC3394311

[28] Colovos C Yeates TO Verification of protein structures: patterns of nonbonded atomic interactions. Protein Sci (1993) 2(9):1511–9. 10.1002/pro.5560020916 8401235 PMC2142462

[29] Wiederstein M Sippl MJ. Prosa-web: interactive web service for the recognition of errors in three-dimensional structures of proteins. Nucleic Acids Res (2007) 35:W407–10. 10.1093/nar/gkm290 17517781 PMC1933241

[30] Budayova-Spano M Grabarse W Thielens NM Hillen H Lacroix M Schmidt M Monomeric structures of the zymogen and active catalytic domain of complement protease C1r: further insights into the C1 activation mechanism. Structure (2002) 10:1509–19. 10.1016/S0969-2126(02)00881-X 12429092

[31] Gaboriaud C Rossi V Bally I Arlaud GJ Fontecilla-Camps JC. Crystal structure of the catalytic domain of human complement c1s: a serine protease with a handle. EMBO J (2000) 19:1755–65. 10.1093/emboj/19.8.1755 10775260 PMC302006

[32] Dobó J Harmat V Beinrohr L Sebestyén E Závodszky P Gál P. MASP-1, a promiscuous complement protease: structure of its catalytic region reveals the basis of its broad specificity. J Immunol (2009) 183(2):1207–14. 10.4049/jimmunol.0901141 19564340

[33] Harmat V Gal P Kardos J Szilagyi K Ambrus G Vegh B The structure of MBL-associated serine protease-2 reveals that identical substrate specificities of C1s and MASP-2 are realized through different sets of enzyme-substrate interactions. J Mol Biol (2004) 342:1533–46. 10.1016/j.jmb.2004.07.014 15364579

[34] Brooks BR Bruccoleri RE Olafson BD States DJ Swaminathan SA Karplus M CHARMM: a program for macromolecular energy, minimization, and dynamics calculations. J Comput Chem (1983) 4(2):187–217. 10.1002/jcc.540040211

[35] Tovchigrechko A Vakser IA GRAMM-X public web server for protein–protein docking. Nucleic Acids Res (2006) 34(Suppl_2):W310–4. 10.1093/nar/gkl206 16845016 PMC1538913

[36] Singh R Purkait B Abhishek K Saini S Das S Verma S Universal minicircle sequence binding protein of *Leishmania donovani* regulates pathogenicity by controlling expression of cytochrome-b. Cell Biosci (2016) 6(1):13. 10.1186/s13578-016-0072-z 26889377 PMC4756535

[37] Presanis JS Hajela K Ambrus G Gál P Sim RB Differential substrate and inhibitor profiles for human MASP-1 and MASP-2. Mol Immunol (2004) 40:921–9. 10.1016/j.molimm.2003.10.013 14725788

[38] Keizer MP Pouw RB Kamp AM Patiwael S Marsman G Hart MH TFPI inhibits lectin pathway of complement activation by direct interaction with MASP-2. Eur J Immunol (2015) 45(2):544–50. 10.1002/eji.201445070 25359215

[39] Morrison LS Goundry A Faria MS Tetley L Eschenlauer SC Westrop GD Ecotin-like serine peptidase inhibitor ISP1 of *Leishmania major* plays a role in flagellar pocket dynamics and promastigote differentiation. Cell Microbiol (2012) 14:1271–86. 10.1111/j.1462-5822.2012.01798.x 22486816 PMC3440592

[40] Roos A Bouwman LH Munoz J Zuiverloon T Faber-Krol MC Fallaux-van den Houten FC Functional characterization of the lectin pathway of complement in human serum. Mol Immunol (2003) 39(11):655–68. 10.1016/S0161-5890(02)00254-7 12493641

[41] Okroj M Holmquist E King BC Blom AM. Functional analyses of complement convertases using C3 and C5-depleted sera. PLoS One (2012) 7(10):e47245. 10.1371/journal.pone.0047245 23071769 PMC3468486

[42] Pietrocola G Rindi S Rosini R Buccato S Speziale P Margarit I The group B *Streptococcus*-secreted protein CIP interacts with C4, preventing C3b deposition via the lectin and classical complement pathways. J Immunol (2016) 196(1):385–94. 10.4049/jimmunol.1501954 26608922 PMC4683360

[43] Ansari MY Equbal A Dikhit MR Mansuri R Rana S Ali V Establishment of correlation between in-silico and in-vitro test analysis against *Leishmania* HGPRT to inhibitors. Int J Biol Macromol (2016) 83:78–96. 10.1016/j.ijbiomac.2015.11.051 26616453

[44] Ansari MY Ahsan MJ Yasmin S Sahoo GC Saini V Das P In silico identification of novel antagonists and binding insights by structural and functional analyses of guanylate kinase of *Leishmania donovani* and interaction with inhibitors. Gene Rep (2017) 8:134–43. 10.1016/j.genrep.2017.07.003

[45] Manas RD Md Yousuf A Sinha S Ali V Roshan Kamal T JyotiPrava M Computational elucidation of novel antagonists and binding insights by structural and functional analyses of serine hydroxymethyltransferase and interaction with inhibitors. Gene Rep (2017) 10:17–25. 10.1016/j.genrep.2017.10.010

[46] Wilkinson KD Quantitative analysis of protein-protein interactions. Methods Mol Biol (2004) 261:15–32. 15064447 10.1385/1-59259-762-9:015

[47] Al-Hamaoy RR AL-Saeed DHH Khudhair MS A new method for the determination of dissociation constant (kd) on the binding of CA19-9 to its antibody in type 2diabetic patients by enzyme linked immunosorbent assay (ELISA) with some modifications. Int J Adv Res (2016) 4:710–9. 10.21474/IJAR01/265

[48] Rogers ME. The role of *Leishmania* proteophosphoglycans in sand fly transmission and infection of the mammalian host. Front Microbiol (2012) 3:223. 10.3389/fmicb.2012.00223 22754550 PMC3384971

[49] Brittingham A Mosser DM Exploitation of the complement system by *Leishmania* promastigotes. Parasitol Today (1996) 12(11):444–7. 10.1016/0169-4758(96)10067-3 15275279

[50] Arnold JN Wormald MR Suter DM Radcliffe CM Harvey DJ Dwek RA Human serum IgM glycosylation: identification of glycoforms that can bind to mannan-binding lectin. J Biol Chem (2005) 280:29080–7. 10.1074/jbc.M504528200 15955802

[51] Cestari IDS Evans-Osses I Freitas J Inal JM Ramirez MI. Complement C2 receptor inhibitor trispanning confers an increased ability to resist complement-mediated lysis in *Trypanosoma cruzi*. J Infect Dis (2008) 198(9):1276–83. 10.1086/592167 18781865

[52] Norris KA. Stable transfection of *Trypanosoma cruzi* epimastigotes with the trypomastigote-specific complement regulatory protein cDNA confers complement resistance. Infect Immun (1998) 66(6):2460–5. 9596703 10.1128/iai.66.6.2460-2465.1998 PMC108225

[53] Punetes SM Da Siva RP Sacks DL Hammer CH Joiner KA. Serum resistance of metacyclic stage *Leishmania major* promastigotes is due to release of C5b-9. J Immunol (1990) 145(12):4311–6. 2147941

[54] Hermoso T Fishelson Z Becker SI Hirschberg K Jaffe CL Leishmanial protein kinases phosphorylate components of the complement system. EMBO J (1991) 10:4061–7. 1756717 10.1002/j.1460-2075.1991.tb04982.x PMC453154

[55] Nunes AC Almeida-Campos FR Horta MF Ramalho-Pinto FJ. *Leishmania amazonensis* promastigotes evade complement killing by interfering with the late steps of the cascade. Parasitology (1997) 115(Pt 6):601–9. 10.1017/S0031182097001704 9488871

[56] Joshi PB Kelly BL Kamshawi S Sacks DL Mc Master WR. Targeted gene deletion in *Leishmania major* identifies leishmanolysin (GP63) as a virulence factor. Mol Biochem Parasitol (2002) 120(1):33–40. 10.1016/S0166-6851(01)00432-7 11849703

[57] Perona JJ Craik CS Evolutionary divergence of substrate specificity within the chymotrypsin-like serine protease fold. J Biol Chem (1997) 272:29987–90. 10.1074/jbc.272.48.29987 9374470

[58] Kastritis PL Bonvin AM On the binding affinity of macromolecular interactions: daring to ask why proteins interact. J R Soc Interface (2013) 10:20120835 10.1098/rsif.2012.0835 23235262 PMC3565702

[59] Gaboriaud C Gupta RK Martin L Lacroix M Serre L Teillet F The serine protease domain of MASP-3: enzymatic properties and crystal structure in complex with ecotin. PLoS One (2013) 8:e67962. 10.1371/journal.pone.0067962 23861840 PMC3701661

[60] Ambrus G Gal P Kojima M Szilagyi K Balczer J Antal J Natural substrates and inhibitors of mannan-binding lectin-associated serine protease-1 and -2: a study on recombinant catalytic fragments. J Immunol (2003) 170:1374–82. 10.4049/jimmunol.170.3.1374 12538697

[61] Megyeri M Mako V Beinrohr L Doleschall Z Prohaszka Z Cervenak L Complement protease MASP-1 activates human endothelial cells: PAR4 activation is a link between complement and endothelial function. J Immunol (2009) 183:3409–16. 10.4049/jimmunol.0900879 19667088

[62] Mishra A Antony JS Gai P Sundaravadivel P Van TH Jha AN Mannose-binding lectin (MBL) as a susceptible host factor influencing Indian visceral leishmaniasis. Parasitol Int (2015) 64(6):591–6. 10.1016/j.parint.2015.08.003 26297290

[63] Green PJ Feizi T Stoll MS Thiel S Prescott A McConville MJ. Recognition of the major cell surface glycoconjugates of *Leishmania* parasites by the human serum mannan-binding protein. Mol Biochem Parasitol (1994) 66(2):319–28. 10.1016/0166-6851(94)90158-9 7808481

[64] Hajishengallis G Lambris JD. Crosstalk pathways between toll-like receptors and the complement system. Trends Immunol (2010) 31:154–63. 10.1016/j.it.2010.01.002 20153254 PMC2849859

[65] Hawlisch H Belkaid Y Baelder R Hildeman D Gerard C Kohl J. C5a negatively regulates toll-like receptor 4-induced immune responses. Immunity (2005) 22(4):415–26. 10.1016/j.immuni.2005.02.006 15845447

[66] Hawlisch H Kohl J. Complement and toll-like receptors: key regulators of adaptive immune responses. Mol Immunol (2006) 43:13–21. 10.1016/j.molimm.2005.06.028 16019071

[67] Strainic MG Liu J Huang D An F Lalli PN Muqim N. Locally produced complement fragments C5a and C3a provide both costimulatory and survival signals to naive CD4+ T cells. Immunity (2008) 28(3):425–35. 10.1016/j.immuni.2008.02.001 18328742 PMC2646383

[68] Ruhland A Kima PE. Activation of PI3K/Akt signaling has a dominant negative effect on IL-12 production by macrophages infected with *Leishmania amazonensis* promastigotes. Exp Parasitol (2009) 122:28–36. 10.1016/j.exppara.2008.12.010 19186178 PMC2669696

[69] Kima PE PI3K signaling in *Leishmania* infections. Cell Immunol (2016) 309:19–22. 10.1016/j.cellimm.2016.09.004 27622385 PMC5127740

[70] Asad MD Ali N Dynamicity of immune regulation during visceral leishmaniasis. Proc Indian Natn Sci Acad (2014) 809:247–67. 10.16943/ptinsa/2014/v80i2/55105

[71] Kopf M Brombacher F Köhler G Kienzle G Widmann KH Lefrang K. IL-4-deficient Balb/c mice resist infection with *Leishmania major*. J Exp Med (1996) 184(3):1127–36. 10.1084/jem.184.3.1127 9064329 PMC2192785

[72] Waki H Yamauchi T Kamon J Kita S Ito Y Hada Y Generation of globular fragment of adiponectin by leukocyte elastase secreted by monocytic cell line THP-1. Endocrinology (2005) 146:790–6. 10.1210/en.2004-1096 15528304

