# Supplementary material for: Leishmania donovani Inhibitor of Serine Peptidases 2 Mediated Inhibition of Lectin Pathway and Upregulation of C5aR Signaling Promote Parasite Survival inside Host
Source: Front Immunol. 2018 Jan 29;9:63. doi: 10.3389/fimmu.2018.00063 (PMC5796892; doi:10.3389/fimmu.2018.00063)
Supplement: Supplementary file 1 [file Data_Sheet_1.PDF]

Supplementary Information (SI)

SI Material and methods

Complement mediated killing assay

According to Cestari et al. (8) complement mediated lysis assay was performed to see the effect of normal human serum (NHS) on *L. donovani* viability. Briefly, ~1.0 × 10^7 logarithmic promastigote forms of parasites were re-suspended in 100 µL of fresh RPMI-1640 medium. Cells were treated with NHS or heat inactivated serum (56°C for 30 min) which was pre diluted in different concentration (3.12 %, 6.25%, 12.5%, 25%, 50%, 100%) in RPMI 1640 medium. Cells were kept at 37 °C for 1 hr and after incubation 800 µL of cold RPMI was added and placed on ice. Heat inactivated NHS was used as a negative control. Parasites of each group were stained with trypan blue and quantified in a Neubauer chamber under a light microscope. Viable parasites were counted from each group.

ELISA: Complement activation by *Leishmania* parasites

The comparative analysis of the activation of different compliment pathways by *Leishmania* parasites was detected by ELISA. Activation of the pathways was performed by the assessment of C3b deposition and immune complex MAC (C5b-9) formation. For the activation of individual pathway the other pathways were blocked by using pathway specific inhibitor. For the CP activation, LP and AP were blocked by anti human MBL antibody and factor B antibody respectively. For the LP activation, C1q and factor B antibody was used to block CP and AP respectively. For the AP activation assay EGTA and C1 inhibitor treated serum was used. All the dilution of the serum was prepared in VBS^{2+} as discussed previously. *Leishmania* cells (~10^7) was first washed three times with ice cold Gelatin, HEPES buffer saline containing 2 mM $CaCl_2$ and 0.5 mM $MgCl_2$ (GHB^{2+}) for CP and LP assay. For CP activation ELISA plate was coated with human IgM as discussed previously. Processed parasites (~ 10^7/ml) were fixed in 4% paraformaldehyde and added in IgM coated ELISA plate with binding buffer and incubate for overnight at 4 °C (11). Similarly, for LP and AP activation parasites were fixed in 4% paraformaldehyde and coated in ELISA plate with coating buffer for overnight at 4 °C. The wells were washed three times with phosphate buffered saline (PBS, 137 mM NaCl, 2.7 mM KCl, 4.3 mM Na_2HPO4, 1.47 mM KH_2PO4)/ 0.05% tween 20, then blocked with 3% bovine serum albumin (BSA) for 2 hours at RT. 100 µl of heat inactive human serum (1:10 v/v) or different pathway specific serum (1:10 v/v) was added to the wells and incubated at 37 °C for 1 hr. Complement activation via each group was detected by assessment of C3b deposition and C5b-9 complex formation by using

anti C3b antibody and C5-9 complex antibody followed by incubation with HRP conjugated secondary antibody. The experiments were performed in triplicate and data are means ± SD from three separate experiments. An asterisk (*) denotes P≤0.05, double asterisk (**) denotes P≤0.001 when compared to control.

Macrophage neutrophil elastase activity

Neutrophil elastase activity was measured in macrophages cells and their culture derived supernatant. Briefly, THP1 cells were maintained in density of ~1×10^{7} in RPMI media as discussed previously.THP1 cells were washed with PBS and plated in 6 well plates in density of ~2×10^{6} cells/ well in serum free RPMI 1640 containing 100 ng/ml PMA. After 12 hr of incubation the medium was collected and centrifuged at 1500 rpm for 15 minutes at 4 °C. Supernatant was collected and concentrated according to Waki H et al. (52). Simultaneously, the cells were extracted and protein sample was prepared. Cell protein extract or supernatant used for NE activity assessment were either left untreated or treated with NE inhibitor ecotin or rLdISP2 (1 to 10 µM). Activity was measured in buffer containing 100 mM Tris-HCl, pH 8.0, 2.5% (v/v) dimethyl sulfoxide (DMSO) and incubated in ice for 30 minutes. Subsequently, the appropriate chromogenic substrate i.e. N-methoxy succinyl–Ala-Ala-Pro-Val-7-amino-4-methyl curamin was added in treated and untreated NE. Enzymatic hydrolysis of the substrate was monitored by spectrofluorometer by measuring the release of fluorescence (excitation 380 nm, emission at 460 nm). All the experiments were performed in triplicate and the data expressed as means ± SD from three independent experiments.

TABLES

Table 1 *Leishmania donovani* ISP1 and ISP2 interaction with C1r and C1s

**C1r and ISP1**			
**Interaction**	**Distance**	**Donor**	**Acceptor**
C1r:LEU561:HN - ISP1:HIS56:NE2	2.23598	HN	NE2
C1r:ARG650:HH11 - ISP1:GLN57:O	2.1309	HH11	O
C1r:ARG650:HH12 - ISP1:GLN57:O	1.50652	HH12	O
**C1r and ISP2**			

Interaction	Distance	Donor	Acceptor
C1r:ASP532:HN - ISP2:PRO106:O	2.27868	HN	O
C1r:TYR535:HH - ISP2:TYR137:O	1.61092	HH	O
C1r:ARG665:HH22 - ISP2:VAL142:O	2.37595	HH22	O
**C1s and ISP1**			
**Interaction**	**Distance**	**Donor**	**Acceptor**
C1s:GLU452:HN - ISP1:TYR36:OH	2.13715	HN	OH
C1s:LEU535:HN - ISP1:ILE134:O	2.35597	HN	O
C1s:SER540:HN - ISP1:PHE103:O	1.99998	HN	O
C1s:SER540:HG - ISP1:PHE103:O	2.18713	HG	O
C1s:LYS631:HZ2 - ISP1:CYS136:O	2.30135	HZ2	O
C1s:LYS631:HZ3 - ISP1:CYS136:O	2.38269	HZ3	O
C1s:GLN665:HE22 - ISP1:TYR132:OH	1.94812	HE22	OH
ISP1:CYS136:HN - C1s:CYS534:SG	2.20375	HN	SG
ISP1:CYS136:HG - C1s:CYS534:SG	2.3649	HG	SG
**C1s and ISP2**			
**Interaction**	**Distance**	**Donor**	**Acceptor**
C1s:LYS579:NZ - ISP2:THR12:OG1	2.92562	NZ	OG1
ISP2:LYS57:N - C1s:GLU506:OE2	2.80924	N	OE2
ISP2:ARG105:NE - C1s:ASP441:O	3.16717	NE	O
ISP2:ARG105:NH2 - C1s:ASP441:O	1.96919	NH2	O
ISP2:ARG136:NH2 - C1s:CYS613:SG	2.87975	NH2	SG
ISP2:ARG136:NH2 - C1s:CYS644:SG	2.00546	NH2	SG
ISP2:TYR137:OH - C1s:GLY645:O	2.72396	OH	O
ISP2:ARG138:NE - C1s:GLU559:O	2.85993	NE	O

Table 2 *Leishmania donovani* ISP1 and ISP2 interaction with MASP1 and MASP2

**MASP1 and ISP1**			

Interaction	Distance	Donor	Acceptor
MASP1:GLN579:HE21 - ISP1:GLN142:OE1	2.22076	HE21	OE1
MASP1:GLN579:HE22 - ISP1:GLN142:OE1	2.17213	HE22	OE1
MASP1:ARG655:HN - ISP1:GLN142:O	2.1545	HN	O
ISP1:GLN142:HE22 - MASP1:GLN579:OE1	1.29744	HE22	OE1
**MASP1 and ISP2**			
**Interaction**	**Distance**	**Donor**	**Acceptor**
MASP1:SER561:HN - ISP2:SER25:OG	2.34324	HN	OG
ISP2:ARG73:HH22 - MASP1:LEU564:O	2.0665	HH22	O
**MASP2 and ISP1**			
**Interaction**	**Distance**	**Donor**	**Acceptor**
MASP2:LYS452:NZ - ISP1:GLN83:O	1.92246	NZ	O
MASP2:LYS503:NZ - ISP1:SER62:OG	2.65903	NZ	OG
MASP2:HIS508:ND1 - ISP1:LEU111:O	2.47149	ND1	O
MASP2:ASN547:ND2 - ISP1:MET106:O	2.98546	ND2	O
ISP1:THR58:OG1 - MASP2:PRO453:O	1.65659	OG1	O
**MASP2 and ISP2**			
**Interaction**	**Distance**	**Donor**	**Acceptor**
ISP2:ALA54:HN - MASP2:THR466:OG1	1.95078	HN	OG1
ISP2:ASN55:HD21 - MASP2:LEU575:O	1.99116	HD21	O
ISP2:ASN55:HD22 - MASP2:LEU575:O	2.26042	HD22	O
MASP2:THR467:HN - ISP2:ALA54:O	2.15633	HN	O
ISP2:ARG54:HH11 - MASP2:ASP594:OD2	2.05136	HH11	OD2

Figures and Legends (SI)

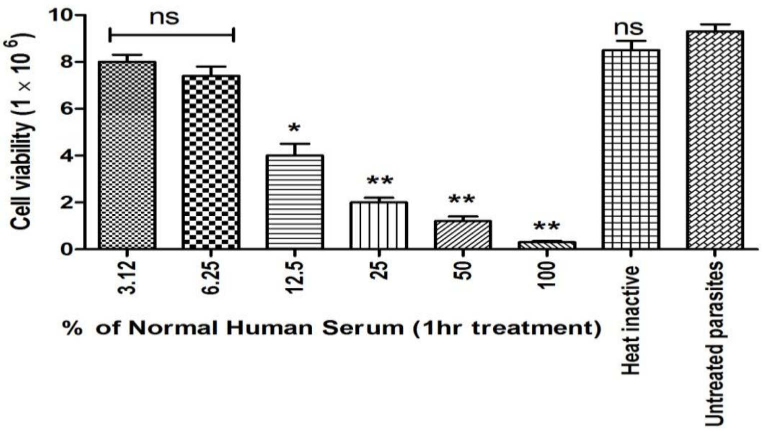

Figure S1 Effect of normal human serum (NHS) on the parasite viability.

Leishmania parasites were either treated with different concentration of NHS or left untreated and incubated at 37 °C for 1hr. Cell viability were measured by trypan blue methods. The experiments were performed in triplicate and data are means ± SD from three separate experiments. An asterisk (*) denotes $P \leq 0.05$ when compared to heat inactive serum treated parasite.

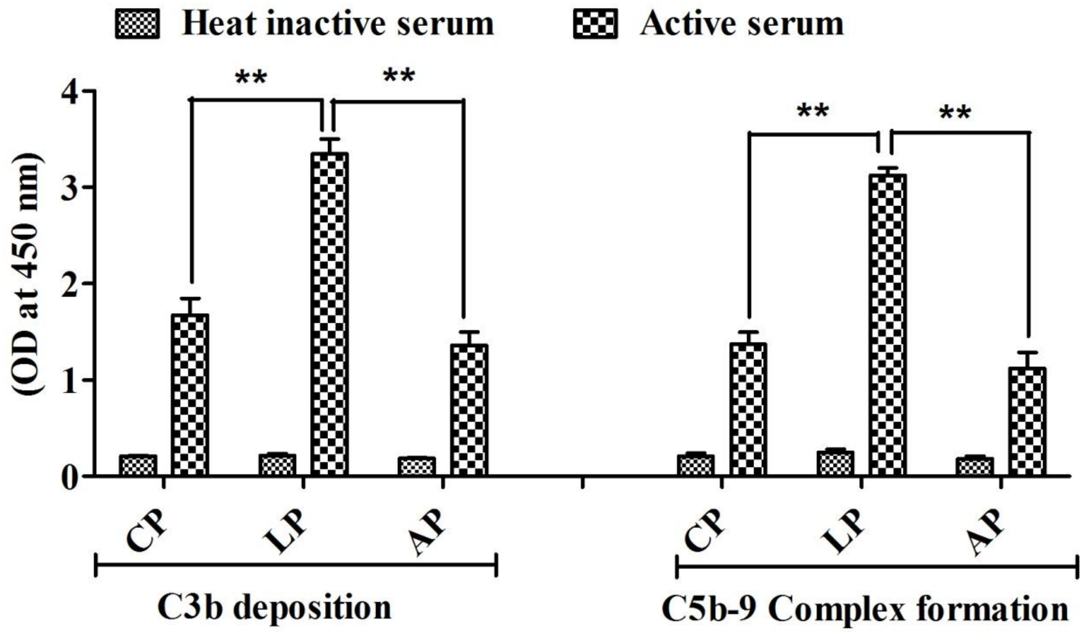

Figure S2 Activation of different complement pathways by *Leishmania donovani*.

ELISA was performed to observe the activation of different complement pathways by Ld parasites. *Leishmania* parasites were processed accordingly as discussed previously, fixed with 4% paraformaldehyde and coated in ELISA plate. Simultaneously, the coated cells were

treated with different pathway specific serum. ELISA plates were incubated with C3b antibody and C5b-9 antibody respectively and formation of C3b and C5b-9 complex was assessed by HRP conjugated secondary antibody. Heat inactivated serum was used as negative control. The experiments were performed in triplicate and data are means ± SD from three separate experiments. An asterisk (*) denotes $P \leq 0.05$ and double asterisk (**) denotes $P \leq 0.001$ when compared to control one.

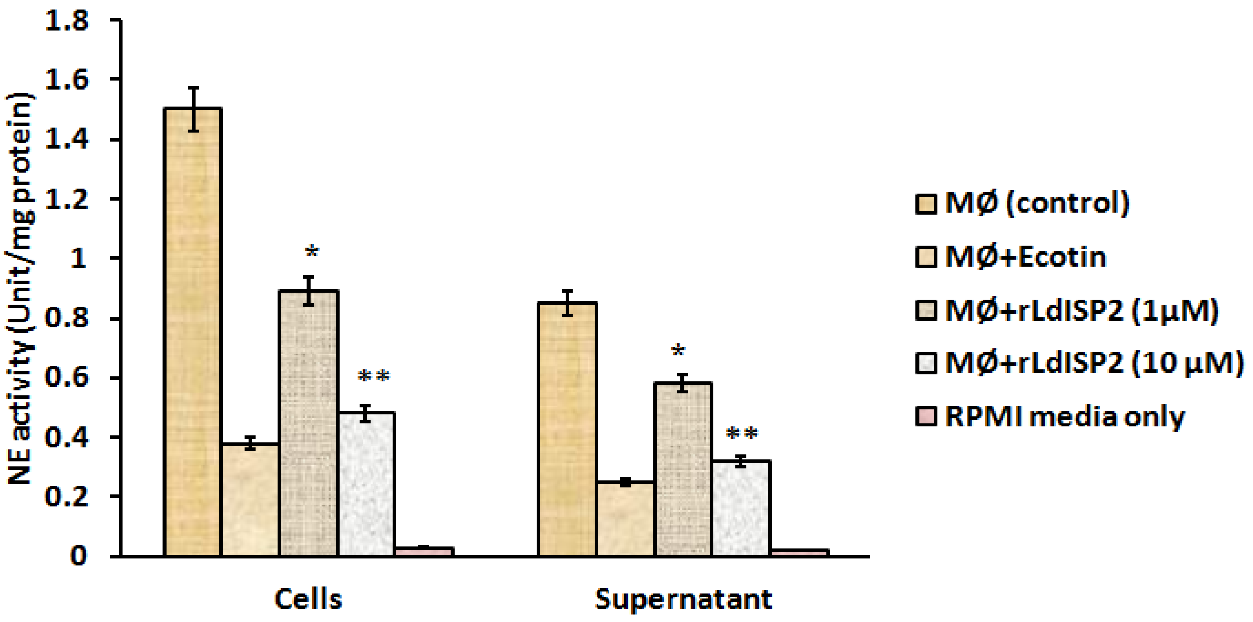

Figure S3 Enzyme activity of macrophages neutrophil elastase

Macrophage cells (~2 × 10^6) extract and culture supernatant were used to measure neutrophil elastase activity. To see the role of inhibitor on NE activity cell extract and supernatant were either left un-treated or treated with neutrophil elastase inhibitors ecotin and rLdISP2 (1 to 10 µM). Simultaneously, appropriate fluorogenic substrate i.e. N-methoxy succinyl–Ala-Ala-Pro-Val-7-amino-4 –methyl curamin (200 µM) was added in treated or untreated protein sample for the assessment of neutrophil elastase activity. Enzymatic hydrolysis of the substrate was monitored by measuring the release of fluorescence (excitation 380 nm, emission at 460 nm) through spectrofluorometer. All the experiments were performed in triplicate and the data expressed as means ± SD from three independent experiments.

Results of SI

Normal human serum was effective in killing the *Leishmania* parasites

From the compliment mediated killing assay we have found the significant reduction in the viability of the parasites with increasing concentration of serum. In serum treated parasites we have observed significant ~53%, ~77%, ~86% and ~96% reduction in the viability of the parasites in 12.5%, 25%, 50% and 100% serum respectively when compared to the untreated parasites. However, no significant difference in the viability of the parasites was observed in presence of 3.12 % and 6.25% serum. Our data clearly indicates that normal human serum is effective in killing the parasites by complement mediated lysis.

Lectin pathway is the most activated pathway by *Leishmania* parasites

We have analysed the comparative activation of different complement pathways (classical, lectin and alternative) by *Leishmania* parasites. The assessment of the activation of different pathway was performed on the basis of C3b formation and thereby membrane attacking complex (C5b-9) formation by individual pathway. C3b formation via lectin pathway was found to be ~2 and ~2.4 fold higher than the classical and alternative pathway respectively. Similarly active C5b-9 complex (MAC) formation was also found to be increased by ~2.3 and ~2.8 fold by lection pathway when compared to classical and alternative pathway respectively. Our data clearly suggested that LP is the most activated pathway by the *Leishmania* parasites rather than the CP and AP.

***Leishmania* LdISP2 inhibits macrophages derived neutrophil elastase activity**

Macrophages derived neutrophil elastase activity was analysed by enzymatic assay. Our data depicted the presence of NE in macrophages cells extract and in culture supernatant. Simultaneously, the inhibitory effect of rLdISP2 on macrophage derived NE was analysed and we have found that macrophages cells extract derived NE activity get significantly decreased by ~1.6 and ~ 3 fold in presence of 1µM and 10 µM of rLdISP2 respectively when compared to control. Similarly, in culture supernatant we have found ~1.4 fold and ~2.6 fold decrease in the NE activity in presence of 1µM and 10 µM of rLdISP2 respectively when compared to control. Our data clearly indicates that presence of LdISP2 reduce the activity of macrophages.
